# Natural products as novel anti-obesity agents: insights into mechanisms of action and potential for therapeutic management

**DOI:** 10.3389/fphar.2023.1182937

**Published:** 2023-06-20

**Authors:** Ummul Fathima Shaik Mohamed Sayed, Said Moshawih, Hui Poh Goh, Nurolaini Kifli, Gaurav Gupta, Sachin Kumar Singh, Dinesh Kumar Chellappan, Kamal Dua, Andi Hermansyah, Hooi Leng Ser, Long Chiau Ming, Bey Hing Goh

**Affiliations:** ^1^ PAPRSB Institute of Health Sciences, Universiti Brunei Darussalam, Gadong, Brunei; ^2^ School of Pharmacy, Suresh Gyan Vihar University, Jaipur, India; ^3^ Department of Pharmacology, Saveetha Institute of Medical and Technical Sciences, Saveetha Dental College and Hospitals, Saveetha University, Chennai, India; ^4^ Faculty of Health, Australian Research Centre in Complementary and Integrative Medicine, University of Technology Sydney, Ultimo, NSW, Australia; ^5^ School of Pharmaceutical Sciences, Lovely Professional University, Phagwara, India; ^6^ Department of Life Sciences, School of Pharmacy, International Medical University, Kuala Lumpur, Malaysia; ^7^ Discipline of Pharmacy, Graduate School of Health, University of Technology Sydney, Ultimo, NSW, Australia; ^8^ Uttaranchal Institute of Pharmaceutical Sciences, Uttaranchal University, Dehradun, India; ^9^ Department of Pharmacy Practice, Faculty of Pharmacy, Universitas Airlangga Surabaya, Indonesia; ^10^ School of Medical and Life Sciences, Sunway University, Sunway, Malaysia; ^11^ Biofunctional Molecule Exploratory Research Group, School of Pharmacy, Monash University Malaysia, Bandar Sunway, Malaysia; ^12^ College of Pharmaceutical Sciences, Zhejiang University, Hangzhou, China

**Keywords:** obesity, medicinal plants, adipogenesis, white adipose tissue, brown adipose tissue, WAT browning

## Abstract

Obesity affects more than 10% of the adult population globally. Despite the introduction of diverse medications aimed at combating fat accumulation and obesity, a significant number of these pharmaceutical interventions are linked to substantial occurrences of severe adverse events, occasionally leading to their withdrawal from the market. Natural products serve as attractive sources for anti-obesity agents as many of them can alter the host metabolic processes and maintain glucose homeostasis via metabolic and thermogenic stimulation, appetite regulation, pancreatic lipase and amylase inhibition, insulin sensitivity enhancing, adipogenesis inhibition and adipocyte apoptosis induction. In this review, we shed light on the biological processes that control energy balance and thermogenesis as well as metabolic pathways in white adipose tissue browning, we also highlight the anti-obesity potential of natural products with their mechanism of action. Based on previous findings, the crucial proteins and molecular pathways involved in adipose tissue browning and lipolysis induction are uncoupling protein-1, PR domain containing 16, and peroxisome proliferator-activated receptor-γ in addition to Sirtuin-1 and AMP-activated protein kinase pathway. Given that some phytochemicals can also lower proinflammatory substances like TNF-α, IL-6, and IL-1 secreted from adipose tissue and change the production of adipokines like leptin and adiponectin, which are important regulators of body weight, natural products represent a treasure trove for anti-obesity agents. In conclusion, conducting comprehensive research on natural products holds the potential to accelerate the development of an improved obesity management strategy characterized by heightened efficacy and reduced incidence of side effects.

## 1 Introduction

Obesity poses a serious threat to worldwide public health and is defined by a Body mass index (BMI) of 30 kg/m^2^ or higher (Lin and Li, 2021). Obese individuals are at risk of developing various chronic diseases, such as diabetes mellitus, hypertension, cancer, and neurological disorders, which could be severely impacted by the buildup of excess body fat (WHO, 2021). According to the World Health Organization (2021), 13% of the adult population worldwide was obese in 2016, a figure that has tripled since 1975. Currently, more than 1.9 billion adults are overweight (with BMI of 25.0–29.9) and more than 650 million are considered obese (Haththotuwa et al., 2020). In Europe, around 23% of women and 20% of men are obese. In Western nations, the prevalence of obesity and type 2 diabetes mellitus (T2DM) are on the rise (WHO, 2022). Decades of research have been devoted to understanding the relationship between obesity and metabolic problems, as well as the connection between obesity and adipose tissue, which is considered a metabolically active endocrine organ (Xu et al., 2003; Wang Q. et al., 2015; Jiang et al., 2020). As a matter of fact, adipose tissue is also involved in other functions such as the regulation of glucose and lipid metabolism, insulin sensitivity, inflammatory response, non-shivering thermogenesis, and vascular endothelial function (Kwok et al., 2016).

A growing body of research suggests that the dysfunction in adipose tissue drives the development of obesity (Sam and Mazzone, 2014). In general, there are two main types of adipose tissue: a) white adipose tissue (WAT) which is widely distributed in the human body, and b) brown adipose tissue (BAT) which is found in the cervical, supraclavicular, axillary, paravertebral, mediastinal, and upper abdominal regions in adult humans (Maurer et al., 2021). Majority of WAT stored in the subcutaneous region in deep and superficial abdominal parts and the gluteal-femoral regions, but there are also some distribution of WAT in the visceral region such as in the omental, mesenteric, mediastinal, and epicardial regions (Sbarbati et al., 2014). Under the skin, subcutaneous WAT serves as a buffer against mechanical stress from the outside world, and an insulator to keep heat in and prevent dermal infection. On the other hand, visceral WAT wraps around internal organs inside the peritoneum and the rib cage (Zwick et al., 2018). The central role of WAT is to store excess energy as triglycerides which is antagonistic to the function of brown adipose tissue (BAT), which dissipates energy by producing heat and warms up the blood supply to vital organs (Saely et al., 2012). The brown appearance of BAT is due to the presence of high mitochondrion content and dense vascularization. Uncoupled protein 1 (UCP-1) is employed in the inner membrane of the mitochondria to help BAT use and dissipate the energy derived from lipids to generate heat (Cannon and Nedergaard, 2004).

In addition to its role as an energy storage organ, WAT plays major role in obesity because these cells secretes unique regulatory substances with endocrine, paracrine, and autocrine functions (Fruhbeck et al., 2001). Several substances secreted by adipocytes play significant roles in various aspects of physiological control. For instance, leptin and adiponectin contribute to body weight regulation, while TNF-α, IL-6, and IL-1β are associated with local inflammation resulting from obesity. Additionally, substances like Ang II and PAI-1 impact vascular function, and estrogens are involved in reproductive processes (Gómez-Hernández et al., 2016). WAT modulates metabolic activities in other peripheral tissues and the brain by secreting adipocytokines such as leptin and adiponectin (Kershaw and Flier, 2004). Leptin, a hormone that is mostly secreted by adipocytes, plays an important role in controlling body weight via its central effects on hunger and peripheral effects on regulating energy expenditure (Marti et al., 1999). Adiponectin is another hormone secreted by adipocytes that regulates food intake. Several investigations have reported hypoadiponectinemia in individuals with obesity, diabetes, and coronary artery disease (Arita et al., 1999). Consequently, restoring the regulatory function of WAT appears to be a viable strategy for combating obesity.

Apart from that, numerous studies have pointed out the presence of a subpopulation of WAT that has adapted characteristics of BAT, such as increased UCP-1 expression, adipocyte locularity, mitochondrion density, and vascularization, is known as brite or beige adipose tissue (Harms and Seale, 2013). The process that involves the browning of WAT or what is termed adaptive thermogenesis is usually triggered by low temperatures. In healthy adult humans, metabolically active adipose tissue depots with beige-like features were found in the cervical, supraclavicular, axillary, and paravertebral regions (Nedergaard et al., 2007). The reprogramming of WAT to BAT or even beige adipose tissue garnered much interest in the scientific community as this conversion constitutes a great opening to tackle obesity by increasing energy expenditure and restoring glucose homeostasis balance (Kuryłowicz and Puzianowska-Kuźnicka, 2020).

As an alternative to conventional treatments against obesity and associated problems, natural products, such as medicinal plants in the form of pure compounds or extracts, are widely accessible on the market (Hasani-Ranjbar et al., 2013). Phytochemicals can exert their anti-obesity effects through different mechanisms such as inhibiting digestive enzyme activities (pancreatic lipase and amylase), appetite regulation, and reducing the formation of WAT or increasing WAT browning (Fu et al., 2016). Moreover, the phytoconstituents found in diverse plants have proven to possess a range of additional mechanisms of actions against obesity, including promoting PPAR-α and PPAR-β expression, suppressing ghrelin, and regulating plasma lipid profile (Raoof and Kareem, 2020). Certain nutritional compounds isolated from fruits, vegetables, and edible plants, such as curcumin from turmeric (Lee et al., 2011), anthocyanins from blueberries, epigallocatechin gallate from green tea and nobiletin from citrus peel, have been found to be useful in treating metabolic diseases (Chan et al., 2021). Typically, these natural substances limit adipose tissue formation by inhibiting adipocyte differentiation and adipogenesis and lowering triacylglycerol levels by boosting lipolysis or decreasing lipogenesis pathways (Sun et al., 2016). The current review aims to an overview of the numerous types of adipose tissues and their specific functions prior to discussing the possibility of natural products to reduce obesity based on their mechanism of action. The current review also presents how certain natural products influence main molecular pathways involved in WAT browning and elucidated action mechanisms that are associated with energy homeostasis and thermogenesis.

### 1.1 Energy balance and thermogenesis by different adipose tissues

Thermogenesis is essential for the survival of homeotherms. At thermoneutrality or 23°C for an adult man, obligatory thermogenesis is sufficient to sustain normal body temperature and function (Silva, 2003). Adaptive thermogenesis, also known as facultative thermogenic mechanisms requiring or not requiring shivering, is initiated when the ambient temperature falls below thermoneutrality (Yau et al., 2020). The shivering thermogenesis responds to cold by contracting the skeletal muscles to generate heat; this boosts the resting metabolic rate by five-fold in humans (Eyolfson et al., 2001), but it cannot be sustained for an extended period of time (Periasamy et al., 2017). In contrast, non-shivering thermogenesis occurs predominantly in BAT, which oxidizes lipids by activating the lipase enzyme and releases energy as heat during prolonged exposure to cold (Cannon and Nedergaard, 2010). Besides the free fatty acids released from triglycerides by lipases via β-oxidation in BAT, β 3-adrenergic receptors (β 3-AR) present in brown adipocytes increase the expression of thermogenic marker Uncoupling protein 1 (UCP-1) protein via protein kinase A (Tan et al., 2011). Upon activation, these UCP-1 proteins in the inner mitochondrial membrane of brown and beige adipocytes translocate protons (H^+^) from the intermembrane space into the mitochondrial matrix. This increases the respiratory chain activity and diminishes the proton motive force utilized by ATP synthase. Due to the conversion of the available energy from substrate oxidation, heat is created (Nicholls, 2006).

### 1.2 Major metabolic pathways in WAT browning

A condition known as “white fat browning” occurs when specific white adipose tissue drastically raise their gene expression and production of proteins such as the UCP-1 protein, thus, giving them the ability to burn fat and produce energy. White adipocyte browning results in the formation of beige also known as brite adipocytes that resemble the brown adipocyte phenotype but are present within the WAT. The conversion of white adipocytes into beige adipocytes may be caused by various stimuli (Bargut et al., 2017). Subcutaneous adipocytes are more likely to undergo browning than visceral adipocytes because they are predominantly smaller and have a greater potential to differentiate (Gustafson and Smith, 2015). UCP-1 protein has key roles in thermogenesis, nonetheless, it depends on the tissue and the type of stimulus. As reviewed previously, UCP-1 protein levels in mitochondria isolated from the “white” adipose depot of cold-induced adipocytes were nearly identical to those in brown-fat mitochondria. The thermogenic function of UCP-1 protein was evidenced by UCP-1-dependent thermogenesis with lipid or carbohydrate substrates by increasing the canonical guanosine diphosphate (GDP) sensitivity. The thermogenic density of WAT measured by UCP-1-dependant oxygen consumption of WAT was one-fifth that of brown adipose tissue, and the overall quantitative contribution of all white-fat mitochondria was one-third that of brown adipose tissue, indicating that the conventional brown adipose tissue depots would still dominant in thermogenesis (Shabalina et al., 2013; Ikeda and Yamada, 2020). Among the widely studied pathways for stimulating the browning of white adipocytes are the actions of norepinephrine, which is released by sympathetic nerve terminals and binds with β-adrenergic receptors mainly β _3_-AR found on the surface of adipocytes to carry out its functions (Otton et al., 2021).

Stimulation of β _3_-AR causes the p38 mitogen-activated protein kinase (p38 MAPK) to stimulate activating transcription factor 2 (ATF-2), thus leading to the transcription of peroxisome proliferator-activated receptor gamma coactivator 1α (PGC-1α) (Robidoux et al., 2005). Later, PGC-1α promotes two pathways leading to the formation of brite adipocytes; mitochondrial biogenesis and peroxisome proliferator-activated receptors (PPAR) activation (Hondares et al., 2011). In mitochondrial biogenesis, the PGC-1α activates nuclear respiratory factor 1 (NRF1), which links the nucleus with the mitochondrion and generates mitochondrial replication through mitochondrial transcription factor A (TFAM) activation (Piantadosi and Suliman, 2006). Whereas the three isoforms of PPAR, α, β, and γ, are involved in the transcription of UCP1 (Barbera et al., 2001). This protein is present in the inner mitochondrial membrane as a thermogenesis effector, indicating that mitochondrial biogenesis is required for inducing brite adipocytes. It was found that thermogenic-activated brite adipocytes have a considerable number of mitochondria throughout their cytoplasm (Rossato et al., 2014; Jeremic et al., 2017). Also, the PPARα and β release fatty acids that have multiple roles such as activating UCP-1 and acting as substrates for UCP-1-mediated thermogenesis (Cannon and Nedergaard, 2004), as well as, modulating the transcriptional control (Villarroya et al., 2007). PPARγ is important for adipocyte differentiation (Wu et al., 1999a). It regulates adipogenesis and genes that are involved in the uptake and storage of FAs in WAT (Hansen and Kristiansen, 2006). Furthermore, PGC-1α is a cofactor for the receptor PPARγ, which is required for adaptive thermogenesis in response to decreasing temperatures and is associated with tissue-specific metabolic pathways in the adaptive response to nutritional and environmental stimuli (Ruschke et al., 2010).

**FIGURE 1:**
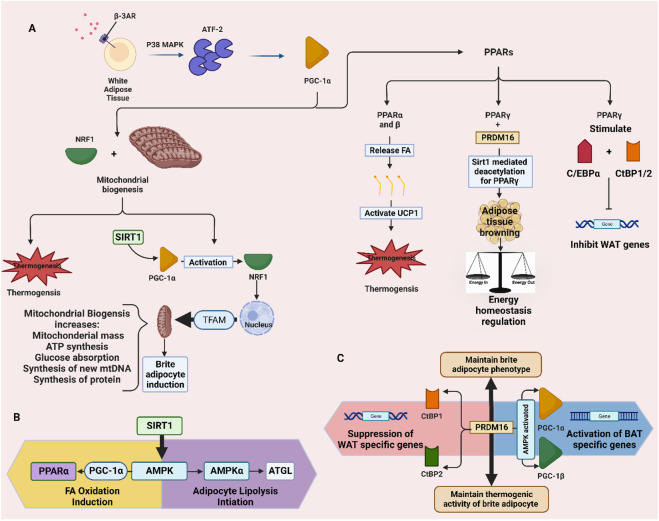
Pathways involved in the browning of white adipose tissue and producing brite adipocytes. **(A)** stimulation of thermogenesis and adipose tissue browning through PPARs and mitochondrial biogenesis. **(B)** SIRT1-AMPK pathway for lipolysis and fatty acid oxidation. **(C)** PRDM16-CtBP1/2 and -PGC-1α and β pathways for suppressing WAT genes and activating BAT genes. ATGL: adipose triglyceride lipase.

Moreover, the commonly agreed pathways that take part in differentiating brite adipocytes from white adipocytes is by detecting the expression of UCP-1 and PR domain containing 16 (PRDM16) (Spiegelman, 2013). It is worth noting that UCP-1 is the protein responsible for thermogenesis, while PRDM16 acts as a stimulus that maintains the brite adipocyte phenotype (Shabalina et al., 2013). It has been shown that at low PRDM16 expression, the brite adipocytes convert back to white adipocytes, thus, PRDM16 is an important molecule for inducing browning and maintaining the thermogenic activity of brite adipocytes (Cohen et al., 2014). The importance of PRDM16 in the browning of subcutaneous white adipocytes is shown by *in vitro* decreasing the amount of small hairpin RNA expressed by PRDM16, which results in a decrease in the expression of thermogenic genes and uncoupled respiration (Seale et al., 2011). The PRDM16 is involved in both the induction of BAT genes and the repression of WAT genes. The induction of BAT-specific genes occurs when PRDM16 binds to the transcriptional coactivators PGC-1α and PGC-1β. Contrarily, the repression of WAT-specific genes is caused by the interaction of PRDM16 with C-terminal binding protein (CtBP) 1 and 2 at the promoter domains of WAT genes (Kajimura et al., 2008). In response to cold, overfeeding in addition to chronic activation of PPARγ, and PRDM16 facilitates the activation of PGC-1α and UCP-1 in WAT by suppressing white fat genes (Seale et al., 2011) (Figure 1).

SIRT1 is a crucial regulator involved in WAT browning (Qiang et al., 2012) and it facilitates erythropoietin production to enhance metabolic activity (Wang et al., 2013). SIRT1 suppresses WAT by inhibiting the nuclear receptor PPARγ (Tamori et al., 2002). PPARγ stimulates the binding of CCAAT/enhancer-binding protein (C/EBPα) and CtBP (1 and 2) and inhibits transcription of WAT-specific genes (Vernochet et al., 2009). It has been reported that browning of subcutaneous WAT is promoted by SIRT1-dependent PPARγ deacetylation via the regulation of ligand-dependent coactivator or corepressor exchange at PPARγ transcriptional complex. Additionally, SIRT1-dependent PPARγ deacetylation is found to regulate energy homeostasis, and promote energy expenditure from energy storage (Qiang et al., 2012). Other than that, SIRT1 promotes threonine phosphorylation, which activates the AMPK signaling pathway and AMPK is known to play a key role in the initiation of adipocyte lipolysis. Besides, PPARα is activated by AMPK via the PGC-1α ligand, which in turn upregulates the gene expression of several key β-oxidation enzymes and promotes fatty acid oxidation (Liu L. et al., 2020). Genes that are responsible for thermogenesis such as UCP1 and PGC-1α, are found to be related by AMPK activation, while SIRT1 was found to promote mitochondrial biogenesis through the activation of PGC-1α (Wu et al., 1999b). In conclusion, the upregulation of SIRT1 mRNA, which reduces PPARγ, was linked to the downregulation of adipogenesis (Feng et al., 2016).

## 2 Pharmacological treatment for obesity

Individuals who are overweight or obese may benefit from bariatric surgery, pharmaceutical treatment, behavioral modifications, and dietary changes, among others. General practitioners and multidisciplinary support teams are vital in assisting patients in losing weight in a healthy, long-term manner. Life expectancy drops by 1 year for every two percentage points increase in its average BMI, accordingly to a modeling study (Semlitsch et al., 2019). Therefore, structured guidelines were developed to create a clinical pathway for the management of overweight and obesity in primary care as reviewed by (Semlitsch et al., 2019). Numerous drugs, such as serotonin receptor agonists have been discovered to be useful in weight loss. In the 1970s, it was revealed that serotonin or 5‐hydroxytryptamine (5-HT) possesses anorectic properties, as heightened brain serotonin levels was associated with increased satiety (Blundell, 1977). For instance, fenfluramine and d-fenfluramine, which are the direct agonists of 5-HT receptors exhibited anti-obesity properties by increasing the release of serotonin in the synaptic cleft. Both compounds modify eating behavior in a manner consistent with satiety (Halford et al., 2005). However, these medications were taken off the market due to arising valvular heart disease (Elliot and Chan, 1997). On the other hand, selective serotonin reuptake inhibitors (SSRIs) could also be administered to increase serotonin levels in obesity treatment by blocking its reuptake into the nerve terminals (Leibowitz et al., 1990). The SSRI fluoxetine decreases food intake and improves satiety feeling (Halford et al., 1998), but it may induce adverse symptoms such as headache, nausea, somnolence, asthenia, diarrhea, sleeplessness, anxiety, sweating, and tremor (Wise, 1992). In addition, 30%–70% of individuals using fluoxetine experienced sexual dysfunction, including erectile dysfunction, anorgasmia, and diminished libido, resulting in non-compliance with the treatment (Colman et al., 2012; Wenthur et al., 2014). Another compound from this class with anti-obesity has been used is sibutramine, which works similarly like fluoxetine as SSRI, but sibutramine also blocks the reuptake of norepinephrine and partially dopamine, both of which have been shown to have anorectic effects *in vivo* (Balcioglu and Wurtman, 2000). However, sibutramine was pull off the market due to the increased risk of cardiovascular disease in obese patients (Czernichow and Batty, 2010).

Differently, rimonabant, a Cannabinoid receptor type 1 (CB1) antagonist has been used in the management of obesity. In response to fasting, the two potent appetite-stimulating hormones, cannabinoid and ghrelin, are known to rise in concentration in the gastrointestinal tract. The administration of CB1 receptor inverse agonist leads to decreased levels of these two hormones, which subsequent resulting in decreased food intake as observed in the 24-h food-starved rats and partially satisfied rats (Gómez et al., 2002). Rimonabant also prevents fat storage in adipocytes by regulating the level of adipose tissue lipoprotein lipase, which is augmented by cannabinoid treatment (Bensaid et al., 2003). Despite that, in Europe and India, rimonabant was withdrawn from the market in 2007 owing to its unwanted psychiatric side effects which included anxiety and depression (Onakpoya et al., 2016).

At the time of writing, orlistat is the only anti-obesity medication that functions independently of the central nervous system and does not enter the bloodstream. In addition, it is the only pancreatic lipase inhibitor treatment that is presently being used in clinical practice (Ballinger and Peikin, 2002). In order to exert its therapeutic effect, it forms a covalent connection with the active serine residue of gastric and pancreatic lipases in the lumen of the digestive system. This action then prevents the hydrolysis of dietary fat (in the form of triglycerides) into absorbable free fatty acids and monoglycerols (Ransac et al., 1991). The side effects associated with orlistat commonly emerge during the initial stages of therapy and tend to attenuate with the progression of treatment. The gastrointestinal tract is primarily affected by these side effects, which can include the presence of oily stool (Liu T.-T. et al., 2020). Another anti-obesity drug that is currently available is liraglutide, which is a glucagon-like peptide‐1 (GLP‐1) receptor agonist. The weight reduction effects of GLP-1 are assumed to be due to appetite suppression and delayed stomach emptying (Jelsing et al., 2012). The common adverse events of liraglutide involve the gastrointestinal tract such as nausea (Burcelin and Gourdy, 2017). In clinical studies, gastrointestinal intolerance was the most frequent reason for liraglutide discontinuation in individuals reported adverse events (Mehta et al., 2017). Meanwhile, exenatide, another GLP-1 agonist approved for obesity treatment, showed a higher reduction in body weight when lifestyle modifications were adopted (Rosenstock et al., 2010).

## 3 Plant-derived natural products as anti-obesogenic agents

Having seen the adverse effects caused by the bulk of the drugs now used for the treatment of obesity, researchers are now turning to natural resources in a quest of compounds with fewer side effects for the management of obesity. Plant-derived natural products have been demonstrated to have anti-obesity effects via a variety of pathways, including metabolic and thermogenic stimulants, appetite regulators, pancreatic lipase and amylase inhibitors, insulin sensitivity enhancers, and adipogenesis inhibitors and adipocytes apoptosis inducers.

### 3.1 Plant-derived natural products as metabolic and thermogenic stimulants

**FIGURE 2:**
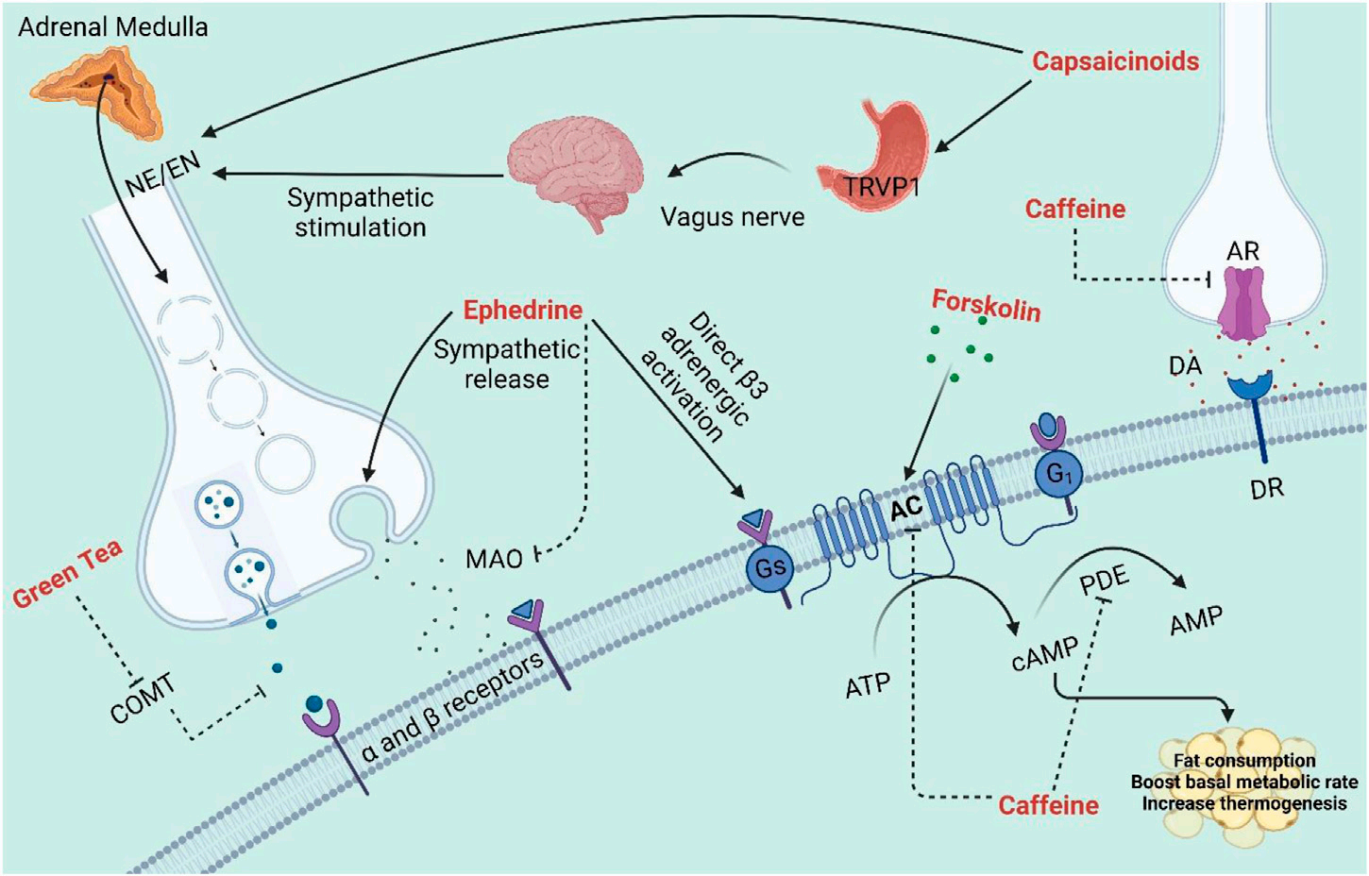
The effects of several NPs on diverse physiological pathways as metabolic and thermogenic stimulants. AC: Adenylyl cyclase; AR: Adenosine receptor; DA: Dopamine; DR: Dopamine receptors; MAO: Monoamine oxidase; PDE: Phosphodiesterase; TAPV1: Transient receptor potential vanilloid-1; NE: Norepinephrine; EN: Epinephrine; COMT: Catechol-O-methyltransferase.

Natural products such as caffeine, ephedrine, capsaicin, and green tea have been suggested for obesity management since they may increase energy expenditure and counterbalance the decrease in metabolic rate that occurs with/after weight loss (Figure 2). Despite being in the same category, the combination of caffeine and ephedrine assists people to lose weight over the long run (Astrup et al., 1992). This is likely due to the fact that two substances inhibit different enzymes that may work synergistically, as the former exerts its effect by inhibiting cAMP degradation caused by phosphodiesterase, while the latter stimulates metabolism by enhancing catecholamine release in the sympathetic nervous system (Diepvens et al., 2007a).

#### 3.1.1 Caffeine from *Coffea arabica*

Caffeine is the most consumed stimulant worldwide due to its various effects and mechanisms of action (Ferreira et al., 2019). In fact, the two most used coffee beans in the genus *Coffea* are *Coffea arabica* L*.* and *Coffea canephora* Pierre. Caffeine has been explored as a possible thermogenic agent for body weight reduction and it may alter thermogenesis by preventing the phosphodiesterase-induced degradation of intracellular cyclic AMP (cAMP) (Diepvens et al., 2007b). In the liver, one of the enzymes under the cytochrome P450 oxidase enzyme family, CYP1A2 metabolizes caffeine into three primary metabolites: paraxanthine (84%), theobromine (12%) and theophylline (4%) (Schwarzschild et al., 2003). Due to the structural similarity, the primary pharmacological action of caffeine is to antagonize adenosine receptors and modulate the purinergic system (Van Dam et al., 2020). There are four types of adenosine receptors highly expressed in the human body, namely, A1, A2A, A2B and A3. A1 and A3 receptors inhibits adenylate cyclase by binding to Gi proteins, whereas A2A and A2B stimulates cAMP production by binding Gs protein (Ortweiler et al., 1985). These receptors have been linked to numerous physiological and pathological processes, including heart rhythm and circulation, lipolysis, renal blood flow, immunological function, sleep regulation, angiogenesis, inflammatory diseases, ischemia-reperfusion, and neurodegenerative disorders (Chen et al., 2013).

#### 3.1.2 Ephedrine from *Ephedra sinica*

Ephedrine is one of the four isomers contained in the shrub known as *Ephedra sinica* which is native to China and Mongolia (Saper et al., 2004). It is a phenylpropylamine protoalkaloid and a sympathomimetic agent that functions as a stimulant and a thermogenic agent (Stohs and Badmaev, 2016). Ephedrine increases energy expenditure and promotes weight loss, as reported in several human studies (Astrup, 2000). The main thermogenic effect of ephedrine is mediated by increasing the sympathetic neuronal release of norepinephrine (NE) and epinephrine (Dulloo, 1993). By inhibiting monoamine oxidase, ephedrine also decreases the breakdown of norepinephrine. The interaction of ephedrine with β-3 adrenergic receptors is implicated in the induction of thermogenesis that promotes breakdown of fats and glucose metabolism modulation (Carey et al., 2015). Similarly, the interactions of ephedrine with β-1 and β-2 adrenergic receptors have also been shown to contribute to some of its thermogenic effects (Liu et al., 1995).

#### 3.1.3 Capsaicinoids from *Capsicum annuum*

Hot red peppers of the species *Capsicum annuum* L. (*Capsicum frutescens*), contain a group of pungent chemicals known as capsaicinoids with capsaicin being the primary pungent component (Barceloux, 2009). According to studies conducted by Reinbach et al. (2010) and Ludy et al. (2012), capsaicin has been found to increase the production of catecholamines, norepinephrine, and epinephrine from the adrenal medulla, which in turn stimulates thermogenesis by acting on adrenergic receptors (Reinbach et al., 2010; Ludy et al., 2012). However, capsaicinoids also have the capacity to modify metabolism through the activation of transient receptor potential vanilloid 1 (TRVP1) receptors, where they are similarly thought to be able to enhance energy expenditure and reduce body fat by boosting catabolic processes in adipose tissues (Yoneshiro and Saito, 2013). Numerous studies in small mice have shown that capsaicin and capsinoids stimulate sympathetically-mediated BAT thermogenesis and decrease body fatness (Saito, 2015). Notably, it has been found that a single intraperitoneal or intragastric dose of capsaicin or capsinoids can increase the whole-body energy expenditure, activates the adreno-sympathetic nervous system, increases BAT temperature, and increase the core temperature which is all produced in hours (Kawada et al., 1986; Ono et al., 2011). Most of these reactions are significantly diminished in mice lacking TRPV1 or by β-adrenergic inhibition (Kawabata et al., 2009). In one study, it was found that red pepper-containing meals resulted in a greater increase in energy expenditure than control meals (Yoshioka et al., 1995). Additionally, research in both human and non-human animals revealed that an increase in thermogenesis is disrupted when a β-adrenergic blocker like propranolol is administered (Kawada et al., 1986), suggesting that capsaicin-induced thermogenesis is probably based on β-adrenergic activation. Capsaicin administration leads to a rise in lipid mobilization and a fall in adipose tissue bulk (Kawada et al., 1986). It is also found to cause WAT browning by activating SIRT’s CaMKII/AMPK-dependent phosphorylation, which stimulates SIRT1. This led to the deacetylation and interaction of proteins to stimulate the browning of WAT in the mouse model (Baskaran et al., 2016).

#### 3.1.4 Forskolin from *Coleus barbatus*

Forskolin is a labdane diterpene isolated from the roots of the *Coleus forskohlii* Briq, belonging to the Labiatae family (Lamiaceae), which is native to India, while *Plectranthus barbatus* and *Coleus forskalaei* (Lamiaceae) thought to be the most prevalent species contains forskolin. (Astell et al., 2013). Forskolin acts directly on adenylate cyclase enzyme, which increases cAMP level and eventually drives the lipolysis or the breakdown of fat in adipose tissues (Litosch et al., 1982). Subsequently, fatty acids released from adipose tissue depot also trigger thermogenesis and an increase in the lean tissue. Overall, forskolin may result in a fat reduction without loss of muscle mass (Godard et al., 2005).

#### 3.1.5 Green tea extracts from *Camellia sinensis*

As the most consumed beverage in Southeast Asia, green tea is the decoction of the plant *Camellia sinensis* L. (belonging to the family Theaceae) (Thitimuta et al., 2017). Consuming green tea and green tea extracts have been shown in several studies to improve thermogenesis and fat oxidation (Dulloo et al., 2000; Diepvens et al., 2007b; Westerterp-Plantenga, 2010a; Türközü and Tek, 2017). The composition of green tea extract responsible for the thermogenic effect includes catechins such as epicatechin, epicatechin gallate, epigallocatechin, and epigallocatechin gallate (EGCG); among which, EGCG is the most prevalent catechins, ranges from 50% to 80% of total catechins (Stohs and Badmaev, 2016). It is hypothesized that catechins, especially EGCG, directly inhibit catechol-O-methyltransferase, an enzyme that breaks down norepinephrine, in order to enhance fat oxidation (Borchardt and Huber, 1975). This transient rise in sympathetic nervous system activity results in elevated catecholamine levels, which may enhance fatty acid mobilization and oxidation (Figure 1). Despite the frequent implications of this mechanism in previous studies, there is no clear evidence to support this theory (Jeukendrup and Randell, 2011). Nonetheless, green tea may regulate the PPAR/FGF21/AMPK/UCP1 pathway, which then enhance thermogenic cells induction by reprogramming the first phase of adipocyte differentiation (Bolin et al., 2020). Furthermore, green tea aqueous extract is shown to promote browning markers in inguinal WAT (Li et al., 2021). In line with this, the aqueous extract of green tea markedly increased PGC-1α activation. This transcriptional activation controls the UCP-1 promoter’s activity which increases thermogenesis, fat consumption, and basal metabolic rate (Boström et al., 2012). It was revealed that EGCG-induced adipogenesis suppression could involve the mitogen-activated protein (MAP) kinase, specifically the extracellular signal-regulated kinases (ERKs) which are activated by growth-related signals (Lin et al., 2005).

### 3.2 Plant-derived natural products as appetite regulators

**FIGURE 3:**
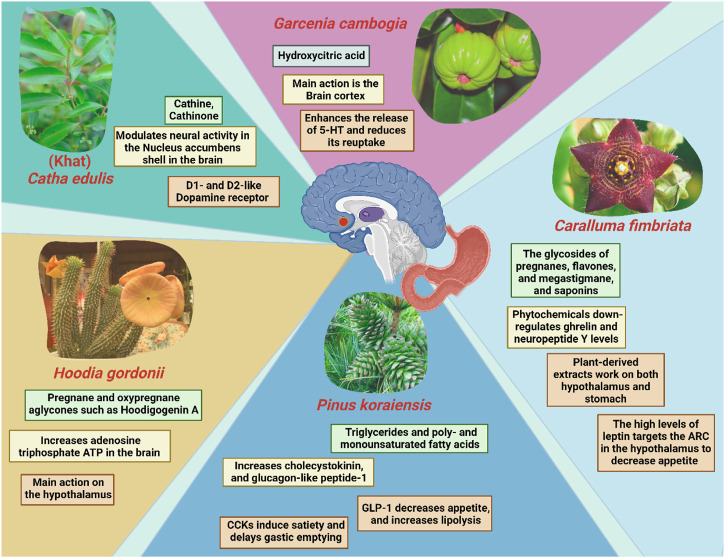
Mechanism of action(s) involved in reducing appetite and/or inducing the feeling of satiety exerted by selected plants and their main chemical constituents. ARC; The arcuate nucleus of the hypothalamus; GLP-1: Glucagon-like peptide-1; CCK: Cholecystokinin.

Food intake is influenced by hunger, satiety, and the physiological mechanisms that balance eating with internal caloric supplies and stable body weight (Sayan and Soumya, 2017). Indeed, anorexic or anorectic agents suppress appetite and reduce body weight, via acting on the satiety centre in the central nervous system, hypothalamus and altering crucial pathways in the neuroendocrine system as well as the brain-gut connection. Various anti-obesity medications including anorexic substances were sold in the past; however, they have all been discontinued due to serious side effects. For instance, the widely used appetite suppressant, sibutramine, was prohibited as anti-obesity agent by Food and Drug Administration and European Medicines Agency due to adverse cardiovascular effects and was discontinued in many countries (James et al., 2010). For further understanding of the mechanism of appetite regulation of pharmacological medicines, it has been found that mild stimulants, such as diethylpropion, phentermine, and bupropion, suppressed food intake, caused weight loss, and modulated neural activity in the nucleus accumbens shell (NAcSh) (Perez et al., 2019), a brain area with strong dopaminergic innervation involved in feeding, sleep, and locomotor behavior (Tellez et al., 2012). It was shown that D1-and D2-like Dopamine (DA) receptor antagonists significantly decreased their anorectic and weight loss effects, contradicting the assumption that they primarily function via norepinephrine and serotonin neurotransmitters (Kalyanasundar et al., 2015). Nonetheless, these drugs are too present some side effects with contraindications for individuals with heart diseases, diabetes, pregnancy, seizure, uncontrolled hypertension, opioid or monoamine oxidase inhibitor use, and may cause vomiting, constipation, dry mouth, or suicidal thoughts (Grunvald et al., 2022). Various phytochemicals aid in inhibiting appetite, with some appetite suppressants having additional benefits such as the browning of WAT that boosts its weight loss efficiency with fewer adverse events encountered as found with the pharmacological intervention (Figure 3).

#### 3.2.1 Khat extracts from *Catha edulis*

Resembling the effect of amphetamine, Khat is a naturally occurring stimulant that is derived from the leaves or young shoots of a flowering plant, *Catha edulis* which is grown in East Africa and the Arabian Peninsula. Although the appetite-suppressing properties of the synthetic amphetamine class stimulants such as pure amphetamine (AMPH) are well known, studies have implicated possibility of the malnutrition and low body mass index due to consumption of these stimulants (Lemieux et al., 2015). To date, very little is known regarding their natural counterpart “khat”. Nevertheless, cultural chewing practice using the leaves of the Khat plant has been known to have appetite-suppressing effects for many years (Halbach, 1972; Zelger and Carlini, 1980). The main active components of *C. edulis* are cathine (D-nor-pseudoephedrine) (NPE) and cathinone (1-aminopropiophenone) (Tucci, 2010). A recent study has supported the idea that NPE-induced food suppression were mediated by dopamine receptors (Fernandes et al., 2020). In another words, NPE has been suggested to have similar anorectic effects as other phenethylamine derivatives like diethylpropion, phentermine, bupropion, and cathinone. This is to no one’s surprise, given that these drugs are all structurally linked to amphetamine and predicted to exert their effects via similar pathways (Khan et al., 2012).

#### 3.2.2 Extracts from Hoodia gordonii

One of the most is *Hoodia gordonii*, commonly known as Bushman’s hat or Kalahari cactus is a popular dietary supplement which is used extensively naturally derived appetite suppressants (Jain and Singh, 2013). It is a succulent plant that is native to Namibia and South Africa and is a member of the Apocynaceae family (Bruyns, 2005). According to a study by Shukla and team, *H. gordonii* contains large amounts of pregnane, oxypregnane, and steroidal glycosides (Shukla et al., 2009). Numerous oxypregnane glycosides were retrieved from *H. gordonii*, such as P57AS3, also known as P57, which is notable for its common aglycone Hoodigogenin A (12-O-tigloyl-3, 14-dihydroxy-pregn-5-ene-20-one). Hoodigogenin A is suggested to be the substance that actively suppresses appetite and raises the amount of adenosine triphosphate ATP in hypothalamic neurons that control food intake (MacLean and Luo, 2004; Geoffroy et al., 2011). While consuming *H. gordonii* in powder supplements, tea, and energy bars appears to have the intended impact on appetite and weight loss, this effect may at least in part be a secondary effect of the substantial side effects linked to ingesting the large dosages necessary to reach therapeutic clinical benefit (Smith and Krygsman, 2014).

#### 3.2.3 Extracts from Caralluma fimbriata

*Caralluma adscendens* var. *Fimbriata* (Wall.), also commonly known as *Caralluma fimbriata* (*C. fimbriata*) is well spread in the dry regions of Asia (Dutt et al., 2012). It is an edible succulent cactus that naturally grows throughout India and is a well-known famine food, hunger suppressant, and thirst quencher (Kuriyan et al., 2007). According to a study, pregnane glycosides, flavone glycosides, megastigmane glycosides, bitter principles, saponins, and numerous other flavonoids are the main phytochemical components of *C. fimbriata.* Pregnane glycosides (Bader et al., 2003). In reality, these compounds are abundant in plants of the Asclepiadaceae family and may account for Caralluma’s appetite-suppressing effects (Schneider et al., 1993). Downregulation of ghrelin production in the stomach and neuropeptide Y (NPY) in the hypothalamus is associated with the appetite reduction effects of *C. fimbriata* extract (CFE), however the precise mechanism of action is yet to be understood fully (Gardiner et al., 2005; Komarnytsky et al., 2013). A clinical study was conducted in 140 overweight adults between 20 and 50 years of age to examine the effects of *C. fimbriata* extract on biomarkers of satiety and body composition (Rao et al., 2021a). There was a significant difference in plasma leptin concentration change between subjects who took the extract and those who did not at week 16. Also, subjects received the treatment had significantly reduced calorie intake from baseline, and consequently a lower waist circumference compared to the placebo group. Furthermore, an increased weight, fat mass, android fat mass, BMI, along with higher level of leptin were reported in the placebo group (when compared to baseline), but not those who undergone extract treatment.

#### 3.2.4 Nut oil from pinus koraiensis

Korean pine nuts have a long history of consumption, notably in the Mediterranean and Asia. Fat constitutes over 60% of *Pinus Koraiensis* oil (PNO). The major constituents of PNO includes triglycerides, poly- and monounsaturated fatty acids (PUFAs and MUFAs), such as 4% palmitic acid, 28% oleic acid, 47% linoleic acid, and 14% pinolenic acid) (Wolff et al., 2000). Consumption of Korean pine nut-free fatty acids (FFA) promotes release of the satiety hormone, cholecystokinin (CCK) (Pasman et al., 2008). CCK causes a delay in stomach emptying, which results in an enhanced sensation of fullness and a decrease in appetite. Long-chain fatty acids are more potent than medium-chain fatty acids at triggering the release of the satiety hormone, and PUFAs are more potent than MUFAs (McLaughlin et al., 1998). According to a study, CCK-8 and glucagon-like peptide-1 (GLP-1) levels have been demonstrated to rise after overweight postmenopausal women were given pine nut-free fatty acids (Tucci, 2010). In clinical investigations, Korean PNO reduced caloric consumption in overweight women (Hughes et al., 2008), enhanced the release of satiety hormones, and lowered appetite in post-menopausal overweight women (Pasman et al., 2008).

#### 3.2.5 Hydroxycitric acid from *Garcenia cambogia*

Hydroxycitric acid (HCA), a popular natural weight loss drug derived from the dried fruit rind of the Southeast Asian tree *Garcinia cambogia* (family Guttiferae) (Ohia et al., 2002). The dried fruit rind is also referred to as Malabar tamarind, and it is widely utilized for culinary uses in southern India (Sergio, 1988). HCA is a competitive inhibitor of ATP citrate lyase that catalyzes the additional mitochondrial cleavage of citrate to oxaloacetate and acetyl-CoA (Ohia et al., 2002). Recent studies have shown that oral HCA supplementation (as Super CitriMaxTM, a calcium/potassium salt of 60% HCA that is tasteless, odorless, and extremely water soluble, HCA-SX) is highly bioavailable in human plasma, as determined by a gas chromatography-mass spectrometric approach (Loe et al., 2001). Additionally, HCA (as Super CitriMaxTM, HCA-SX) enhances the release of 5-HT from rat brain cortex slices *in vitro*, allowing researchers to elucidate how HCA might reduce hunger (Ohia et al., 2001). By altering the neuronal absorption of this monoamine, HCA-SX is thought to enhance the release and accessibility of [^3^H]-5-HT from neuronal serotonergic nerve terminals. These results clearly imply that the influence on 5-HT might be the mechanism underlying the appetite reduction and food intake produced by HCA-SX since elevated brain levels of 5-HT are implicated in regulating sleep, mood changes, and appetite suppression (Ohia et al., 2002). This finally leads to weight reduction due to reduced food intake in addition to other mechanisms such as reduction in body fat percentage, triglycerides, cholesterol and glucose levels, and lipogenesis rate.

### 3.3 Plant-derived natural products as pancreatic lipase and amylase inhibitors

α-amylase is one of the digestive enzymes secreted by the pancreas and salivary glands. It is engaged in vital biological functions such as carbohydrate digestion, whereas its activity is inhibited by many crude drugs (Kobayashi et al., 2000). It has been shown that natural α-amylase inhibitors are beneficial in lowering post-prandial hyperglycemia by delaying the breakdown of carbohydrates and, as a result, reducing the absorption of glucose. Reducing post-prandial hyperglycemia inhibits the formation and storage of triacylglycerol by preventing glucose absorption into adipose tissue (Maury et al., 1993). On the other hand, it is generally recognized that pancreatic lipase must first be used to break down dietary fat before it can be directly absorbed from the intestine. Fatty acid and 2-monoacylglycerol are the two primary by-products of pancreatic lipase hydrolysis (Rani et al., 2012a). Based on these facts, it can be a useful strategy to block these digestive enzymes for treating obesity. Notably, there are numerous digestive enzyme inhibitors isolated from plants that have been reported in the literature, including crude saponins from *Platycodi radix* (Han L. K. et al., 2002), tea saponin (Han et al., 2001), licochalcone A from *Glycyrrhiza uralensis* roots (WonLicochalcone et al., 2007), dioscin from *Dioscorea nipponica* (Kwon C. S. et al., 2003) and the leaves of *Nelumbo nucifera* containing phenolic components (Ono et al., 2006a).

#### 3.3.1 Extracts from *Stellaria media*

Commonly referred to as “Chickweed,” *Stellaria medium* (Linn.) Vill. (Caryophylaceae) is a favorite salad herb that is found all across the Himalayas up to an altitude of 4,300 m (Sharma, 2003). It is an edible medicinal species that are high in β-carotenes, γ-linolenic acid, phenols, vitamins, and minerals. This species shows dose-dependent inhibitory action against pancreatic α-amylase and lipase. However, lipase was more strongly inhibited by *Stellaria media* than α–amylase (Rani et al., 2012a). Generally, *S. media* may reduce the buildup of fat in adipose tissue caused by a high-fat diet by preventing the intestinal absorption of dietary fat and carbohydrates through the inhibition of both enzymes (Rani et al., 2012a).

#### 3.3.2 Extracts from *Achyranthes aspera*

*Achyranthes aspera* Linn (Amaranthaceae), commonly known as apamarga, is an herb that grows abundantly in India on roadsides and waste places. Traditionally, this plant is used as an antimalarial, antileprotic, purgative, diuretic, emmenagogue, oestrogenic, antiarthritic, antispasmodic, cardiotonic, antibacterial, and antiviral agent (Goyal et al., 2007). Oleanane-type triterpenoid saponins have been reportedly found in the extracts of *A. aspera* (EAA) seeds (Hariharan and Rangaswami, 1970) that have elucidated anti-microbial (Peter Amaladhas et al., 2013), wound-healing and anti-inflammatory activities (Hareshbhai, 2021). A study conducted by Rani et al. (2012) showed the effect of phenols, flavonoids, and saponins of *A. aspera* in reducing weight by inhibiting lipases and amylases. The *in vitro* assays employing EAA showed dose-dependent inhibition of lipase and α-amylase activity. EAA inhibited lipase more potently than α-amylase, with an IC_50_ value of 2.34 mg/mL and 3.83 mg/mL respectively. Latha et al. evaluated the hypolipidemic activity of the saponin extract of EAA at 1,200 mg/kg body weight in male Wister rats fed on a high-fat diet for 8 weeks (Latha, 2011). The result demonstrated that when comparing EAA-treated rats to HF diet-only fed rats, a significant reduction was seen in the food efficiency ratio, body weight gain, visceral organ weight indices, serum total cholesterol, triglycerides, very low-density lipoproteins, low-density lipoproteins, atherogenic index, and hepatic total cholesterol and triglyceride levels. When compared to the HF diet alone fed group, the EAA-treated group showed a significant increase in blood high density lipoproteins, fecal total cholesterol, and triglyceride levels. Comparatively, another study administered EAA to mice at a dosage of 900 mg/kg body weight following an oral administration of olive oil, which dramatically reduced postprandial lipid levels at 3 and 4 h (Rani et al., 2012b). When EAA was fed to mice over an extended period of 6 weeks, the blood parameters significantly changed, with lower levels of total cholesterol, total triglycerides, and LDL cholesterol, but higher levels of HDL cholesterol (Rani et al., 2012b). In both of the aforementioned studies, 5-weeks old male Swiss albino mice were employed for the *in vivo* models to determine the pancreatic amylase and lipase inhibitory activity in using similar experiments. In other words, the anti-obesity effects of *A. aspera* were most likely attributed to the anti-oxidant power delivered by saponins and more studies would be helpful to understand other phytochemicals extracted in different parts of this plant.

#### 3.3.3 Extracts from *Nelumbo nucifera*

*Nelumbo nucifera* Gaertn. Is a traditional Chinese herb commonly called lotus, that is widely distributed throughout Eastern Asia. Interestingly, all its plant parts including its fruits, leaves, rhizomes, and seeds are edible and have been used for different purposes including obesity management (Mukherjee et al., 2009). Diverse phytochemical groups were isolated from *N. nucifera* such as alkaloids, flavonoids, megastigmanes, vitamins, and elemanolide sesquiterpenes. Two main actions were inhibited by using *N. nucifera* leaves: a) pancreatic lipases and b) T3-L1 preadipocytes differentiation. The inhibition of pancreatic lipases seems to be mediated by benzylisoquinoline alkaloids, such as trans-N-coumaroyltyramine and trans-N-feruloyltyramine. Besides that, T3-L1 preadipocytes differentiation was strongly inhibited by alkaloids like roemerine oxide, and liriodenine. Other megastigmanes and flavonoids have also been described to significantly reduce fat accumulation but with lower efficacy compared to the above-mentioned ones. On the other hand, due to the presence of an epoxy moiety in their structures, the two compounds; 5,6-epoxy-3-hydroxy-7-megastigmen-9-one and annuionone D strongly suppressed adipocyte differentiation, indicating the significance of the epoxy moiety for the antiadipogenic activity of megastigmanes (Ahn et al., 2013).

#### 3.3.4 Extracts from *Dioscorea nipponica* makino

*Dioscorea nipponica* Makino is a perennial herb belonging to the Dioscoreaceae family that is mostly found in northeastern, northern, eastern, and central China. The Miao and Meng ethnic groups of China have traditionally used this herb’s rhizome to treat pain. Saponin and sapogenins in addition to the phenanthrenes that were extracted from the aerial parts are mainly responsible for most of the pharmacological effects of this plant (Ou-Yang et al., 2018). Saponins glycone and aglycone, namely, dioscin and diosgenin, were isolated from *D. nipponica.* Both compounds suppressed the increase in blood triacylglycerol level in a time-dependent manner when orally injected with corn oil to mice, demonstrating their inhibitory potential against fat absorption. Additionally, during an 8-week study, Sprague-Dawley rats fed a high-fat diet that also contained 40% beef tallow and 5% *D. nipponica* Makino grew much less body weight and adipose tissue than the control animals (Kwon C.-S. et al., 2003).

#### 3.3.5 Extracts from Platycodi Radix

The root of *Platycodon grandiflorum* known as Platycodi Radix is a rich source of saponins (platycosides) which exhibits potent biological activities. It has been used as a traditional oriental medicine and many biological benefits were attributed to the Platycodi Radix extracts (Ha et al., 2006). Example use of Platycodi Radix as a food and a folk remedy includes bronchitis, asthma and pulmonary tuberculosis, hyperlipidemia, diabetes and inflammatory diseases. The saponins extracted from Platycodi Radix was employed in preventing hypercholesterolemia and hyperlipidemia (Lee and Jeong, 2002). Han et al. (Han L.-K. et al., 2002) discussed the effects of crude saponins isolated from Platycodi Radix on fat storage induced in mice by feeding a high fat diet. A high fat diet supplemented with 10 or 30 g/kg crude saponins reduced adipose tissue gain and hepatic steatosis. However, the oral administration of 375 mg/kg saponins in a lipid emulsion inhibited the increases in triacylglycerol blood levels in rats compared with that of rats which did not receive the saponin extracts. Consequently, it was determined that the anti-obesity effect of the crude saponins in mice fed a high fat diet including the reduction of blood triacylglycerol may be due to the inhibition of intestinal absorption of dietary fat by platycodin D. In a different study, 10 known triterpenoidal saponins were purified from Platycodi Radix, among them; platycodin A, C, D, and deapioplatycodin D, as all of them showed intestinal absorption inhibition of dietary fats mediated by pancreatic lipase inhibition (Xu et al., 2005).

#### 3.3.6 Licochalcone A from Glycyrrhiza uralensis

**FIGURE 4:**
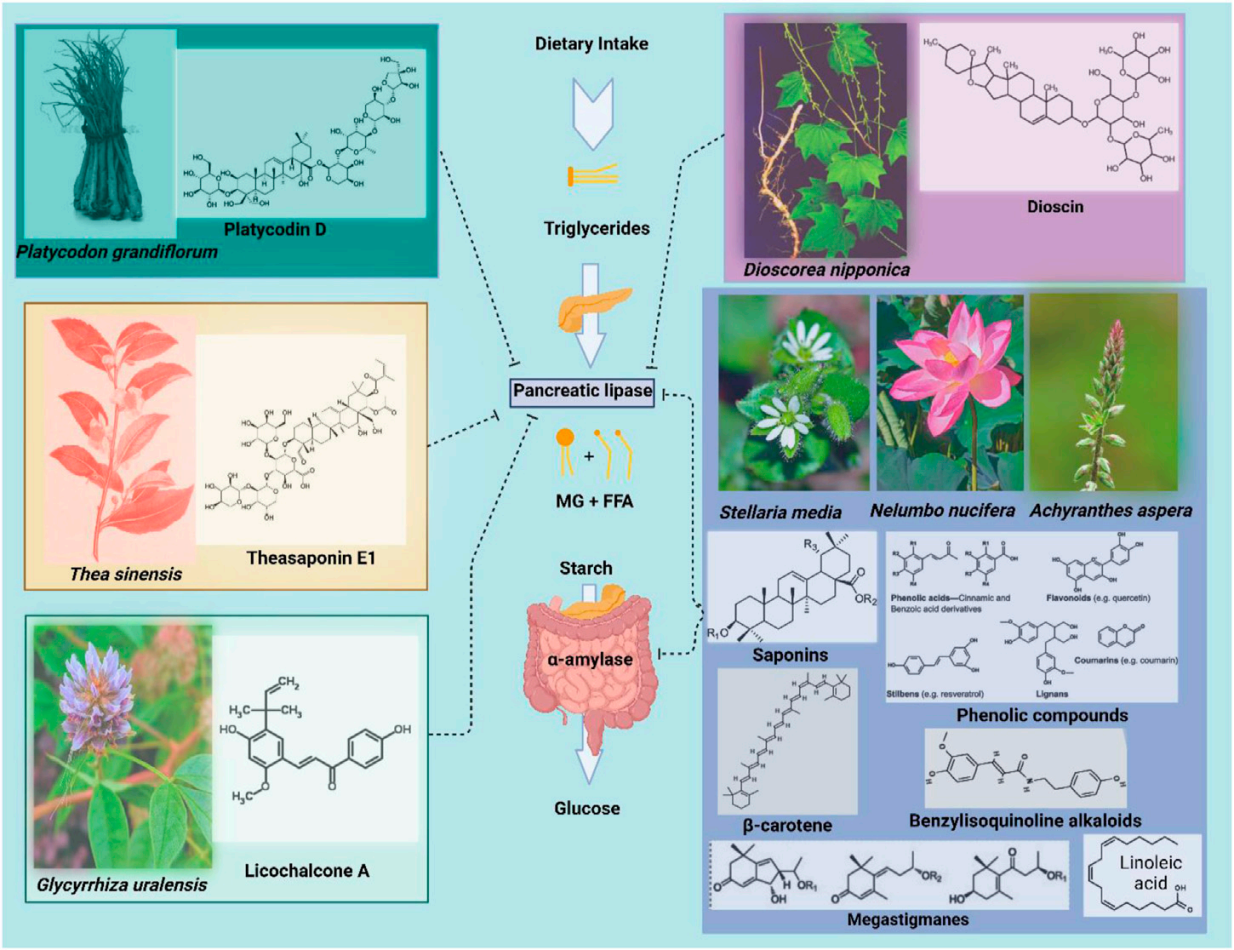
Examples of few plant species with the main phytochemicals and/or groups that inhibit lipases and amylases. MG: monoglycerols, FFA: free fatty acids.

*Glycyrrhiza uralensis* (Fisher) belongs to the family Leguminosae and is widely prized for its therapeutic capabilities, which include antiviral and anti-tumor benefits. These effects are typically attributed to its main bioactive component, glycyrrhizin. In addition, other phytochemical classes isolated from the roots of this plant such as polysaccharides, triterpenes, and flavonoids were reported have anti-inflammatory, anticancer, and antioxidant activities (Afreen et al., 2006; Aipire et al., 2020). In 2007, Won and team purified Licochalcone A from the ethyl acetate/n-hexane fraction of the ethyl acetate extract of *G. uralensis* roots (WonLicochalcone et al., 2007). The team subsequently uncovered that licochalcone A inhibited pancreatic lipase activity reversibly and non-competitively with a Ki value of 11.2 μg/mL (32.8 μM). The same study has also reported pancreatic lipase inhibition mediated the production of oleic acid with both artificial substrate 2,4-dinitrophenyl butyrate and the natural substrate triolein. Intriguingly, licochalcone A extracted from *G. uralensis* increased the browning of inguinal white adipose tissue population in obese mice in addition to inducing the expression of UCP-1 in 3T3-L1 adipocytes (Lee et al., 2018). Figure 4.

### 3.4 Plant-derived natural products improve insulin sensitivity and induce hypoglycemia

Insulin resistance is directly associated with obesity and physical inactivity (Ginsberg, 2000). Adipose tissue is a highly insulin-responsive organ that significantly affects both glucose and lipid metabolism (Luo and Liu, 2016). Furthermore, the adipose tissue of people with obesity and insulin resistance is characterized by a progressive infiltration of macrophages, which may increase the secretion of proinflammatory adipokines, resulting in a progressive failure of adipocyte function, the emergence of insulin resistance and eventually T2DM (Goossens, 2008). There are several studies demonstrated the effects of natural products in diabetes management by improving insulin sensitivity and reducing its resistance. The hypothesis postulated to interpret insulin resistance that eventually leads to diabetes is related to inflammation, mitochondrial dysfunction, and hyperinsulinemia. Other active factors in this mechanism are oxidative stress, endoplasmic reticulum stress, genetic factors, aging, and fatty liver. A more plausible interpretation is cell energy surplus signaled by the adenosine monophosphate-activated protein kinase (AMPK) signaling pathway that indicates high ATP production inside cells. Consequently, effective therapies for reducing obesity-associated insulin resistance pass through one of the following approaches: suppressing ATP production or stimulating their utilization, which can be achieved by restricting calories and exercising. Also, medicines and natural products that sensitize cells eventually inhibit ATP production in mitochondria (Ye, 2013). These products sensitize cells to insulin, mainly targeting PPARγ, which controls several genes that affect the metabolism of glucose and lipids. Those agents improve insulin resistance in humans, specifically by boosting the disposal of insulin-stimulated glucose from skeletal muscle. Besides, individuals with T2DM have illustrated an increase in insulin-stimulated (insulin-resistant substrate) IRS-1-associated PI3K and Akt activity in their skeletal muscle (Choi and Kim, 2010).

#### 3.4.1 Extracts from Trigonella foenum-graecum

Fenugreek *Trigonella foenum-graecum* L. originated in India and North America and was also reported in ancient Egypt and Rome for embalming mummies and facilitating labor and delivery (Smith et al., 2003). The chemical constituents extracted from fenugreek include steroidal sapogenins such as diosgenin, furostanol glycosides, alkaloids such as trigocoumarin, nicotinic acid, trimethyl coumarin, and trigonelline (Wani and Kumar, 2018). In diabetic patients, fenugreek extract has reduced insulin resistance and improved blood glucose management (Gupta et al., 2001). Its anti-hyperglycemic actions have been linked to potentiating insulin release and improving insulin sensitivity (Puri et al., 2002), in addition to preventing intestinal carbohydrate digestion and absorption (Hannan et al., 2007). The anti-hyperglycemic properties are thought to be caused by steroids, saponins, alkaloids, and fiber in the fenugreek seeds (Snehlata and Payal, 2012). According to a clinical study on 18 individuals, fenugreek seed soaked in hot water significantly lowered fasting blood glucose, triglycerides, and very low-density lipoprotein cholesterol (VLDL-C) levels (Kassaian et al., 2009). In another study, when total fenugreek saponins and sulfonylureas were used together as a therapy, 46 patients with type II diabetes witnessed an improvement in clinical symptoms as compared to 23 controls. The combined treatment reduced blood sugar levels (Lu et al., 2008). Besides, trigonelline displayed great potential in treating obesity as it is involved in the browning of 3T3-L1 white adipocytes. The administration of trigonelline resulted in a considerable upregulation of the expression of BAT signature proteins, including PGC-1α, PRDM16, and UCP1 as well as the genes that encode these proteins (Ppargc1α, Prdm16, and Ucp1) in 3T3-L1 white adipocytes (Choi et al., 2021).

#### 3.4.2 Extracts from *Allium sativum*

Garlic (*Allium sativum*) belongs to the Liliaceae family and contains a variety of chemicals, such as organic sulfur compounds, amino acids, vitamins, and minerals. Some organosulfur garlic components are allicin which is very unstable and rapidly decomposes into other sulfur compounds, including ajoene, dithiins, allyl methyl trisulfide, diallyl sulfide, diallyl disulfide, and diallyl trisulfide (Zhang Y. et al., 2020). S-allyl cysteine and diallyl disulfide may have therapeutic benefits on blood glucose, lipid profile, and insulin levels (Agarwal, 1996). The effects of garlic in treating hyperglycemia are investigated by conducting different studies; where in one study, the results showed that garlic decreased serum fructosamine, triglycerides, and fasting blood glucose levels in a 4-week double-blinded placebo-controlled study with 60 T2DM patients (Sobenin et al., 2008). In another study, it was found that in T2DM patients, garlic showed anti-hyperglycemic and anti-hyperlipidemic properties (Ashraf et al., 2005). The mechanisms of garlic are thought to improve hyperglycemia by increasing insulin secretion and enhancing insulin sensitivity (Liu et al., 2005). A randomized study demonstrated that aged garlic extract plus supplement (AGE-S) decreased homocysteine white epicardial adipose tissue (EAT). AGE-S was also found to increase brown EAT and the ratio of brown EAT to white EAT, which was related to the increases in vascular function measured by temperature rebound (Ahmadi et al., 2013). Balogun et al. studied the effect of garlic scape extract in regulating adipogenesis and lipogenesis in white adipose tissue. On the molecular and genetic level, 3T3-L1 cells treated with the extracts exhibited reduced PPAR-γ, CCAAT/enhancer-binding protein a and b, acetyl-CoA carboxylase, fatty acid synthase, sterol regulatory element binding protein 1c, diacylglycerol acyltransferase 1, and perilipin-1 genes. Furthermore, it was also found that lipid accumulation significantly decreased in cells treated during pre-adipogenesis and post-differentiation but less so in cells treated during adipogenesis and differentiation. Additionally, when cells were exposed to garlic scape extract during differentiation, phosphorylation on AMPK and its downstream proteins increased along with elevated levels of carnitine palmitoyl transferase-1α and hormone-sensitive lipase (Balogun and Kang, 2022). In parallel, the β-carboline alkaloid; (1R,3S)-1-methyl-1,2,3,4-tetrahydro-β-carboline-3-carboxylic acid, was the active compound isolated from garlic has suppressed the differentiation of adipocytes in the 3T3-L1 preadipocytes by preventing the remodeling of cytoskeleton, which is required for adipogenesis to take place (Baek et al., 2019).

#### 3.4.3 Extracts from *Hypericum perforatum* L.

*Hypericum perforatum* L., also known as St. John’s wort, has been used for many purposes by the public to treat anxiety, depression, insomnia, gastritis, hemorrhoids, wounds, and burns. (Tokgöz and Altan, 2020). The main bioactive compounds in *Hypericum perforatum* L. are hypericin, hyperforin, and adhyperforin (Gray et al., 2000). It was demonstrated that *H. perforatum* L. extract (EHP) inhibited the protein tyrosine phosphatase 1B (PTP1B) by reducing the gene expression and the catalytic activity of the enzyme (Tian et al., 2015). Insulin resistance and lipid metabolic disorders have been linked to PTP1B, the enzyme that negatively regulates the insulin and leptin signaling pathways (Sun et al., 2007; Basavarajappa et al., 2012). PTP1B inhibitors increase insulin sensitivity by increasing the activity of insulin and leptin receptors (Picardi et al., 2008). Thus, it was found that the treatment with EHP improves hyperinsulinemia, hyperglycemia, insulin tolerance, and glucose infusion rate in the hyperinsulinemic-euglycemic clamp test (Tian et al., 2015).

#### 3.4.4 Extracts from Zingiber officinale

Ginger is the subterranean stem of *Zingiber officinale* Roscoe, which belongs to Zingiberaceae and most likely originated from Southern China. The primary bioactive ingredients are found to be phenolic and terpene compounds such as gingerols that, under heat and prolonged storage converted to shogaols which are transformed by hydrogenation to paradols (Stoner, 2013; Khandouzi et al., 2015). Gingerol treatment enhanced the adipocyte differentiation in mice and improved insulin sensitivity and glucose uptake; thus, it is anticipated that this may also help the diabetic condition (Sekiya et al., 2004). In an *in vivo* study, animals fed a high-fat diet who had their diets enriched with 2% ginger had considerably higher blood insulin concentrations and greater glucose tolerance (Islam and Choi, 2008). Moreover, ginger nanoparticles were fed with HFD for a year to the mice to examine their power to prevent insulin resistance by restoring homeostasis of the transcription factor *Foxa2* in the gut epithelia (Kumar et al., 2022). The ginger nanoparticle feeding boosted *Foxa2* protein expression and protected it from Akt-1-mediated phosphorylation and the subsequent inactivation of *Foxa2*, opposite to HFD, that suppressed its expression. Furthermore, compared to gingerols and 6-shogaol, gingerenone A had a stronger inhibitory effect on adipogenesis and lipid accumulation in 3T3-L1 preadipocyte cells. Additionally, gingerenone A may alter fatty acid metabolism *in vivo* by activating AMPK, that minimize diet-induced obesity. The peroxisome proliferator-activated receptor δ (PPAR-δ)-dependent gene expression in cultured skeletal muscle myotubes was increased by 6-shogaol and 6-gingerol, which enhanced cellular fatty acid catabolism. Also, a randomized, double-blind, placebo-controlled study showed a reduction in BMI among female participants when they took 2 g of ginger powder daily (Mao et al., 2019).

#### 3.4.5 Extracts from *Crocus sativus*

*Crocus sativus* Linn is a member of the Iridaceae family, and its dry stigmas have traditionally been used as a spice or culinary ingredient (Kang et al., 2012). Saffron has a long history of traditional use as a medicinal agent in addition to being a food coloring and aromatic spice. It appeared in ancient writings of prominent scientists like Avicenna as he noted its significant therapeutic effects (Javadi et al., 2013). Saffron extracts include several potent carotenoids, including crocin, and its aglycone crocetin, the monoterpene glycoside picrocrocin, and safranal, which give them pharmacological activities on a diverse range of diseases (Rios et al., 1996). A randomized, double-blind, placebo-controlled clinical trial supported the idea of saffron in treating diabetes, in which it was found that type II diabetes patients who took 100 mg/day of saffron powder for 8 weeks lowered the fasting blood glucose and TNF-α serum levels, along with downregulation of TNF-α and IL-6 mRNA expression (Mobasseri et al., 2020a). Additionally, Milajerdi and team conducted a randomized, triple-blind study involving 54 T2DM patients, and discovered that saffron supplementation daily for 8 weeks significantly lowered the individuals’ fasting blood glucose levels (Milajerdi et al., 2018). Similarly, another placebo-controlled randomized clinical study by Sepaphi found that daily 15 mg of oral crocin administration significantly decreased Hb_A1C_ in diabetic patients compared to the control placebo groups (Sepahi et al., 2018). The underlying mechanisms of action of saffron in diabetes treatment are believed to be insulin sensitivity enhancement, stimulation of insulin signaling pathways, improvement of β-cell activities, promotion of glucose transporter type 4 (GLUT-4) expression, regulation of oxidative stress, repression of inflammatory pathways (Mobasseri et al., 2020b).

#### 3.4.6 Extracts from the genus *Panax*

There are several different species of ginseng, all of which are members of the Aaraliaceae plant family. Ginseng from Korea, Japan, and North America belong to the genus *Eleutherococcus*, while Siberian ginseng belongs to the genus *Panax* (Vogler et al., 1999). Ginsenosides, a broad group of steroidal saponins, are primary ingredients in ginseng that target a wide range of tissues and elicit various pharmacological effects. Their capacity to separately target multireceptor systems at the plasma membrane and activate intracellular steroid receptors may explain some of their complex pharmacological effects (Attele et al., 1999)*.* In several animal models of T2DM, different parts of ginseng (e.g., roots, stems, leaves, and berries) have displayed substantial antihyperglycemic effects. Clinical investigations suggested that ginseng arises as an alternative treatment for T2DM (Vuksan et al., 2000) as it can effectively reduce insulin resistance and fasting blood glucose (Ma et al., 2008). Ginseng possibly reduces blood glucose by increasing insulin production, preserving pancreatic islets, increasing insulin sensitivity, and stimulating glucose uptake (Xie et al., 2011). In particular, by promoting the AMPK pathway, ginseng and ginsenosides decrease energy intake while increasing energy expenditure. Moreover, a study found that black ginseng significantly elevated the expression of brown adipocyte markers (UCP1, PRDM16, and PGC-1) in both types of adipocytes, 3T3-L1 cells and primary white adipocytes. In the same study, administration of the ginsenoside Rb1 enhanced the expression of the brown adipocyte markers in a dose-dependent way in 3T3-L1 cells and primary white adipocytes (Park S. J. et al., 2019). Finally, improving insulin sensitivity and browning the WAT by ginseng chemical constituents can increase the capabilities to reduce weight and prevent obesity.

#### 3.4.7 Essential oils from the genus *Cinnamomum*

Cinnamon is a spice from the Lauraceae family made of the bark of plants from the genus *Cinnamomum* including *Cinnamomum zeylanicum* and *Cinnamomum cassia*. The main active ingredients of cinnamon are the volatile oils extracted from *C. zeylanicum,* and *C. cassia*’s bark, leaves, and roots. Due to the different chemical compositions of distinct plant parts, the pharmacological effects of these volatile oils also vary. Even so, there are few common monoterpene hydrocarbons present in these oils across the three plant parts (i.e., bark, leaves and roots) but in various ratios. The predominant component of the root-bark oil is camphor, while eugenol and cinnamaldehyde present in the leaf oil and bark oil, respectively. Particularly, four oils were extracted from the dried stem bark of *C. cassia*; cinnamaldehyde, cinnamic acid, cinnamyl alcohol, and coumarin (Jayaprakasha and Rao, 2011).

In addition to its traditional and culinary uses, cinnamon has anti-inflammatory, antibacterial, antioxidant, and anticancer properties (Cao et al., 2008; Hariri and Ghiasvand, 2016). Cinnamon enhances insulin sensitivity and has advantageous effects on metabolism (Couturier et al., 2010). In a randomized, controlled clinical trial, cinnamon decreased hemoglobin A1c (Hb_A1C_) by 0.83% compared to conventional therapy alone, which decreased Hb_A1C_ by 0.37% in individuals with T2DM (Crawford, 2009). Furthermore, cinnamon is also described to promote the browning of white adipose tissue by Kwan and team when they observed that subcutaneous adipocytes derived from obese mice models underwent browning post-cinnamon extract treatment. The WAT browning mechanism was marked by an increase in the expression of UCP1 and other brown adipocyte markers in 3T3-L1 adipocytes as well as subcutaneous adipocytes. Additionally, the cinnamon extract treatment increased mitochondrial protein biogenesis (Kwan et al., 2017).

#### 3.4.8 Capsaicin from *Capsicum annuum*

**FIGURE 5:**
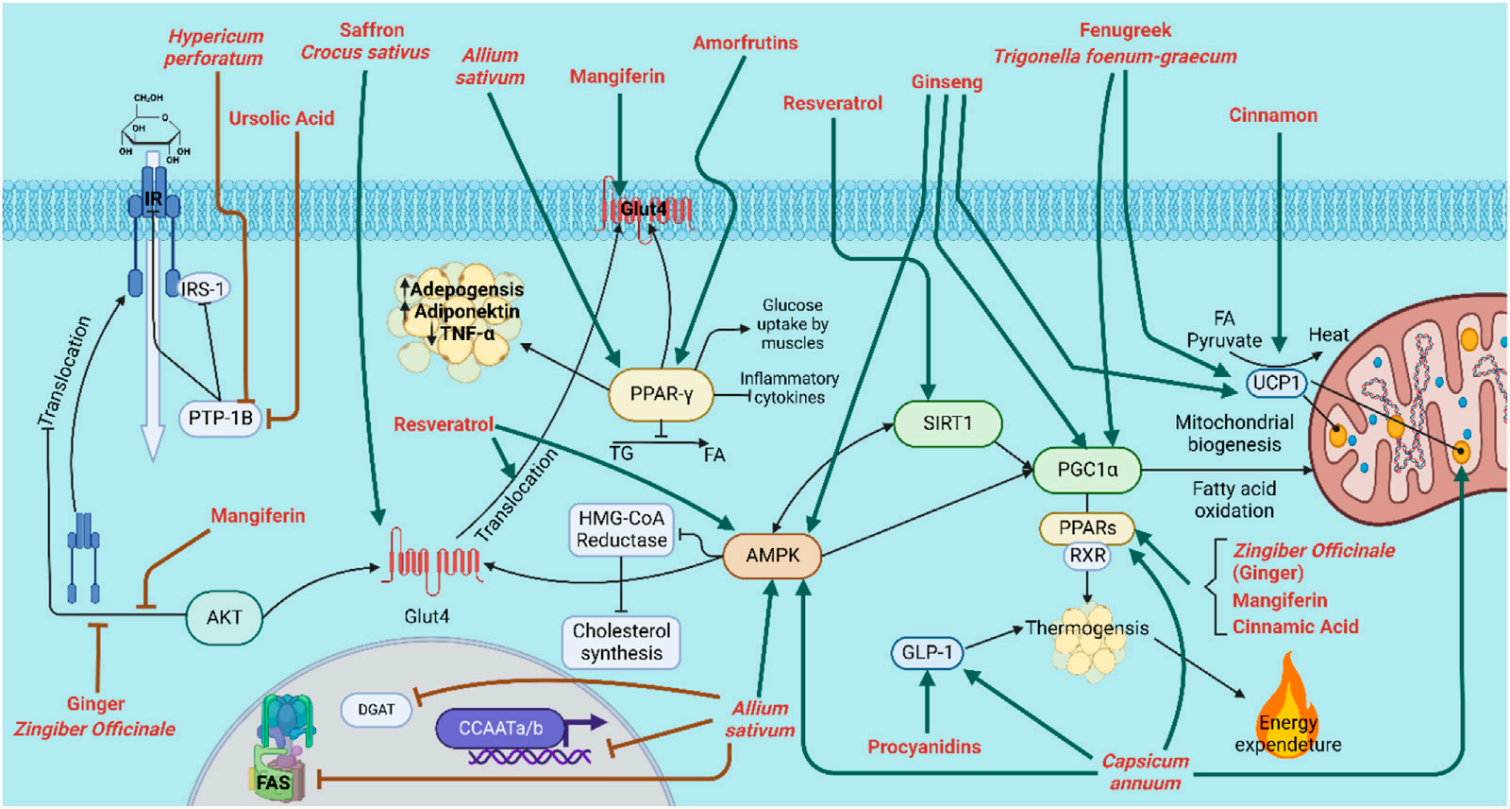
Natural products/plant species that enhance insulin resistance and reduce obesity. IR: insulin receptor; IRS-1: insulin receptor subunit-1; Glut4: Glucose transporter type 4; DGAT: Diglyceride acyltransferase; CCAATa/b: CCAAT/enhancer-binding protein a and b; FAS: Fatty acid synthase; Thick green arrow: increase; Thick red inhibition arrow (ꓕ): inhibition.

The active ingredient in chili peppers, capsaicin, is often taken as a spice. It acts as an agonist on vanilloid channel 1’s transient receptor potential (TRPV1) (Saito, 2015). The receptor plays a significant role in the modulation of metabolic syndrome, where insulin resistance and obesity are present and increase the risk of developing cardiovascular disease, T2DM, and non-alcoholic fatty liver disease (Panchal et al., 2018). *In vitro* and pre-clinical studies have shown low-dose dietary capsaicin to be useful in reducing metabolic problems. Capsaicin’s activation of TRPV1 protein receptor can subsequently regulate adipocyte thermogenesis, and the activation of metabolic modulators such as AMP-activated protein kinase (AMPK), PPARα, UCP1, and GLP-1 (Panchal et al., 2018). Capsaicin enhances insulin sensitivity, increases fat oxidation and reduces body fat; all of which are known to be beneficial for liver and heart health. It was also reported to be effective in diabetic neuropathy and has anti-inflammatory and anti-diabetic properties (Aryaeian et al., 2017) (Figure 5). Capsaicin was also found to decrease lipid accumulation by decreasing PPARγ, C/EBPα, and leptin protein expression, but increased adiponectin expression in 3T3-L1 adipocytes (Hsu and Yen, 2007).

#### 3.4.9 Ursolic acid

The well-known pentacyclic triterpene ursolic acid (3β-hydroxy-12-urs-12-en-28-oic acid) is frequently utilized in traditional Chinese medicine (Bacanli et al., 2019). The primary sources of ursolic acid include *Malus pumila, Ocimum basilicum, Vaccinium* spp.*, Vaccinium macrocarpon, Olea europaea, Origanum vulgare, Rosmarinus officinalis, Salvia officinalis, and Thymus vulgaris* (Ikeda et al., 2008). In diabetic rats, it was demonstrated that ursolic acid (0.05% w/w) altered blood glucose levels and enhanced insulin sensitivity and glucose intolerance. It has also been proposed to preserve pancreatic β-cells and thus increase insulin levels (Jang et al., 2009). Ursolic acid inhibits protein tyrosine phosphatase 1B (PTP1B), an enzyme linked to the downregulation of the insulin receptor, hence increasing the number of insulin receptors and the number of active receptors; it is the mechanism presented in an *in vitro* study, explaining the hypoglycemic activity of ursolic acid (Jung et al., 2007).

#### 3.4.10 Cinnamic acid

The most common sources of cinnamic acid are cinnamon (*C. cassia* L.) J. Presl, citrus fruits, grapes (*Vitis vinifera* L.), tea (*Camellia sinensis* L.) Kuntze, chocolate (*Theobroma cacao* L.), spinach (*Spinacia oleracea* L.), celery (*Apium graveolens* L.), and brassica vegetables (Adisakwattana, 2017). Cinnamic acid (3-phenyl-2-propenoic acid) is an antioxidant phenolic molecule (Bacanli et al., 2019). Cinnamic acid and its derivatives effectively treat diabetes and its complications, among other biological activities. In particular, the impact of cinnamon bark extract on diabetic mice was investigated by Kim et al. (Kim and Choung, 2010). Through the regulation of PPAR-mediated glucose and lipid metabolism, cinnamon extract has been proposed to improve hyperglycemia and hyperlipidemia, increase insulin sensitivity, and lower blood and hepatic lipids. The objective of Lee et al. (Lee et al., 2022) study was to assess the impact of cinnamic acid on obesity, along with its effects on peripheral and hypothalamic inflammation, metabolic profiles, and macrophage-related inflammatory responses in mice fed a high-fat diet (HFD). Results indicated that cinnamic acid-supplement feed reduced obesity and its associated symptoms, such as epididymal fat accumulation, insulin resistance, glucose intolerance, and dyslipidemia, without causing hepatic or renal damage. Additionally, cinnamic acid reduced HFD-induced fat deposition, tumor necrosis factor-α, and macrophage infiltration in the liver and adipose tissue. Moreover, Ly6c^high^ monocytes, M1 adipose tissue macrophages, and hypothalamus microglial activation were all reduced by cinnamic acid. These findings imply that cinnamic acid inhibits the peripheral and hypothalamus inflammatory monocyte/macrophage system and improves metabolic problems associated with obesity.

#### 3.4.11 Resveratrol

Resveratrol (3,4,5-trihydroxystilbene), a polyphenolic compound renowned for its antioxidant and anti-inflammatory attributes, has been identified in several plant species, such as *Polygonum cuspidatum*, *Veratrum grandiflorum*, *V. vinifera*, *Arachis hypogaea*, and *Vaccinium oxycoccos*, among others (Bishayee et al., 2010). Due to its multifaceted mechanisms of action, encompassing enhancements in insulin sensitivity, promotion of GLUT4 translocation, mitigation of oxidative stress, regulation of carbohydrate metabolizing enzymes, activation of crucial signaling pathways mediated by SIRT1 and AMPK, and potential downregulation of adipogenic genes, resveratrol exhibits considerable promise as a therapeutic agent for the treatment of diabetes and other severe diseases (Bagul and Banerjee, 2015). Particularly, through the activation of mitochondrial sirtuin proteins, resveratrol controls blood sugar levels. These proteins regulate the metabolism of sugar and fat; therefore, they significantly impact the body’s ability to produce energy at various levels. This is achieved by enhancing the thermogenesis processes, which increase energy expenditure by burning more adipose tissue (Moshawih et al., 2019). Consequently, resveratrol has the potential to decrease target organ failure and comorbidities that are related to diabetes (Figure 5).

#### 3.4.12 Procyanidins

Procyanidins are the primary flavonoids, also known as flavan-3-ols or flavanols, under proanthocyanidins or condensed tannins family (Aron and Kennedy, 2008). They are oligomeric structures formed by polymerizing 2 to 10 subunits of the monomeric flavanols catechin and epicatechin. Procyanidins are available in different fruits, vegetables, legumes, grains, and nuts such as *Prunus domestica*, *Prunus salicina, Malus domestica, V. vinifera, Prunus amygdalus, Cicer arietinum,* among others (Rue et al., 2018; Bahrin et al., 2022)*.* Few studies have shown the effects of different procyanidins forms on GLP-1. For instance, after being consumed simultaneously with sucrose, berry puree high in proanthocyanidins increased the active GLP-1 levels in healthy adults, leading to a significant decrease in blood glucose levels. Moreover, the administration of another procyanidins derivative, cinnamtannnin A2 increases insulin and active GLP-1 secretion in fasting healthy mice. It was previously described that the L-cells found in the intestine, specifically the ileum and large intestine, and the release of GLP-1 (Törrönen et al., 2012; Yamashita et al., 2013). This hormone is important for controlling glucose homeostasis since its major job is to improve the β-cells’ responsiveness to glucose. It also increases β-cell mass by encouraging proliferation, lowering apoptosis, and boosting β-cell differentiation. It is also that has been found that enteroendocrine cells such as L-cells is a possible target for procyanidin. They have also been reported to reduce the damage caused by the diet, hence enhancing glycemic status and insulin sensitivity in fructose or high-fat-induced insulin resistant models (González-Abuín et al., 2015).

#### 3.4.13 Mangiferin

Mangiferin (MF) is a glucosylxanthone that is present in the mango tree (*Mangifera indica*), the rhizomes of *Anemarrhena asphodeloides* (Miura et al., 2001), and the leaves of *Bombax ceiba*. It is proven to have anti-diabetic, cancer-fighting, antiviral, anti-aging, and antioxidant properties (Dar et al., 2005). Studies revealed that MF increased the AMP-activated protein kinase (AMPK) phosphorylation in 3T3-L1 cells, pancreatic β-cell mass, and the amount of glucose and insulin absorption (Han et al., 2015). To elucidate the mechanism of mangiferin in reducing insulin resistance, Qiao Zhang and team induced lipid accumulation in HepG2 and C2C12 cell lines by using palmitic acid and treated them with various concentrations of MF (Zhang et al., 2019). The outcomes demonstrated that MF significantly increased insulin-stimulated glucose uptake and markedly decreased glucose content in HepG2 and C2C12 cells, as a result of phosphorylated protein kinase B (AKT), GLUT2, and GLUT4 protein expressions. MF also significantly reduced intracellular FFA and triglyceride accumulations and boosted FFA uptake. In HepG2 and C2C12 cells, MF increased the fatty acid oxidation rate corresponding to FFA metabolism, and augmented the activity of PPAR protein and its downstream proteins involved in fatty acid translocase (CD36) and carnitine palmitoyltransferase 1 (CPT1). *In vitro*, oral treatment of mangiferin at a dosage of 20 mg/kg improved insulin sensitivity in Streptozotocin-induced diabetic rats, altered lipid profiles, and reduced the amount of adipokine, and consequently, reduced the inflammation and metabolic syndrome development (Saleh et al., 2014).

#### 3.4.14 Amorfrutins

Amorfrutins are non-toxic components of the fruits of *Amorpha fruticosa*, and the edible roots of licorice, *Glycyrrhiza foetida* (Weidner et al., 2012). The plant *A. fruticosa*, in which the molecules were first discovered, is where the term “amorfrutin” originated. The small, lipophilic amorfrutin class contains the 2-hydroxybenzoic acid as the core structure, and it is surrounded by phenyl and isoprenyl moieties (de Groot et al., 2013). They are a class of natural compounds that have recently been discovered to be PPARγ agonists with selective peroxisome proliferator-activated receptor [γ] modulator (SPPARγMs)-like properties (Lefebvre and Staels, 2012). Amorfrutins are found to be potent PPARγ agonists with a binding affinity range from 236 to 354 nM, in addition to a micromolar affinity for PPARα and PPARβ/δ. It was reported that the affinity of amorfrutin B to the PPARγ receptors is twice as strong as the synthetic anti-diabetic drug pioglitazone (Weidner et al., 2012). The widely targeted type II diabetes protein, PPARγ, is a sensor and regulator that predominates lipid and glucose metabolism and adipose cell differentiation. PPARγ receptor improves insulin sensitivity via various metabolic actions, including adipokines regulation (Malapaka et al., 2012).

### 3.5 Plant-derived natural products inhibiting adipogenesis and inducing adipocytes apoptosis

Adipogenesis is described as transforming preadipocytes into adipocytes by arresting preadipocyte development, accumulating lipid droplets, and producing mature adipocytes with certain morphological and biochemical features (Ghaben and Scherer, 2019; Zhang K. et al., 2020). The initial sign of adipogenesis is a change in cell shape, accompanied by modifications in the type and level of expression of cytoskeletal and extracellular matrix components (Gregoire et al., 1998). These events give rise to the production of two categories of transcriptional factors involved in adipogenesis, namely, C/EBP and PPAR. The main transcriptional regulators of adipogenesis are C/EBPα and PPARγ, which are necessary for producing numerous functional proteins in adipocytes. The terminally differentiated adipocyte phenotype appears predominantly activated and maintained by C/EBPα. At the onset of differentiation, C/EBPβ and C/EBPδ are expressed and thought to control C/EBPα synthesis. The PPARs are part of the nuclear hormone receptor superfamily, which contains retinoid, thyroid, and steroid hormone receptors. For example, activation of PPARα in the liver causes peroxisomes and related enzymes to proliferate, resulting in long-chain fatty acid metabolism via the β-oxidation cycle. Because it is expressed most abundantly in adipose tissue and is activated during the early phase of differentiation of both 3T3-L1 and 3T3-F442A preadipocytes, PPARγ is thought to play a significant role in controlling adipogenesis (Wu et al., 1996).

**FIGURE 6:**
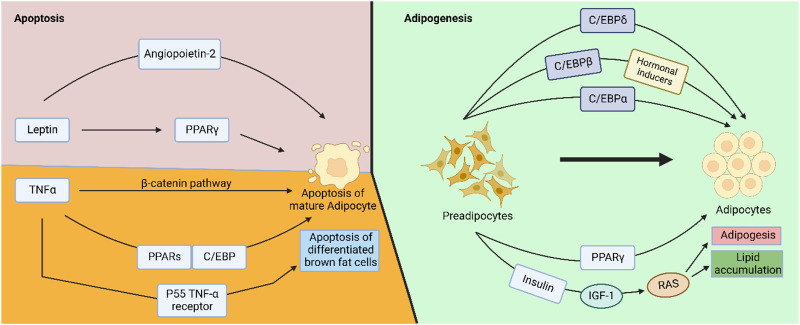
The major molecular pathways in adipocytes’ apoptosis and adipogenesis.

On the other hand, inducing apoptosis is a viable method of removing adipocytes in obese people, which can be achieved through suppressing adipogenesis, obstructing fat accumulation, and deleting adipocytes. Notably, adipokines are the hormones that signal changes in fatty-tissue mass and energy balance to regulate energy consumption. They are secreted from the adipose tissue to control fat weight and homeostasis (Muoio and Newgard, 2005). The two most prominent adipokines that can be considered an excellent strategy to be targeted by natural products are leptin and TNF-α. Leptin is produced and secreted by adipocytes to regulate thermogenesis and food intake through both central and peripheral pathways to increase adipose tissue mass. According to a recent study, adipose tissue in rats that had received leptin by intra-cerebroventricular injection decreased quickly and showed signs of apoptosis. Furthermore, the effect of leptin on peripheral tissue was as efficacious as the centrally administered dose (Zhang and Huang, 2012). To further clarify the mechanism, it has been known that leptin can stimulate the production of angiopoietin-2 in the adipose tissue, which also leads to the development of apoptosis in the cells without increasing the vascular endothelial growth factor. To understand the role of the PPAR-γ in this process, Qian and others analyzed its mRNA levels after intra-cerebrovascular administration. They found that the protein levels increased significantly after leptin treatment, which may indicate its involvement in the mechanism (Qian et al., 1998). TNF-α is the second adipokine generated and produced by adipocytes and plays a vital role in regulating and controlling adipocyte mass and number via apoptotic mechanisms. It has been reported that the P55 TNF-α receptor subtype is responsible for the induction of apoptosis of the differentiated brown fat cells when bound with its substrate, TNF-α. According to a recent study, TNF-α decreases the number of mature adipocytes but not preadipocytes by inducing apoptosis, through C/EBP and PPARγ-mediated inhibition of NF-kB. Moreover, β-catenin pathway was also found to be involved in the TNF-α regulated 3T3-L1 preadipocyte apoptosis (Cawthorn et al., 2007; Tamai et al., 2010). In short, adipogenesis is tightly regulated by neuroendocrine system. At any rate, natural products and phytochemical affecting enzymes or pathways that are related to hunger or satiety can have the potential to indirectly affects adipogenesis and apoptosis induction, and eventually lead to obesity reduction. The following section further explores additional phytochemicals that exert its anti-obesity effects via inhibiting adipogenesis or inducing apoptosis of adipocytes. Figure 6.

Natural substances, including EGCG, genistein, esculetin, berberine, resveratrol, capsaicin, baicalein, and procyanidins, have been shown to inhibit adipogenesis in several studies. In adipocytes treated with genistein, berberine, and rhein, the protein expression of PPAR and C/EBP was lowered (Rayalam et al., 2008). The isoflavone, genistein was first discovered and isolated from the plant *Genista tinctoria*, or dyer’s broom (Mukund et al., 2017), has been demonstrated to reduce PPARγ expression by activating the Wnt/β-catenin pathway and inhibiting C/EBPβ, while it also regulates PPARγ transcriptional activity by activating AMPK during adipogenesis. This shows that genistein may be a potent anti-obesity agent. Differently, berberine (BBR) is the primary component in Cortidis rhizome and has an anti-bacterial activity utilized in Chinese medicine. The mechanism of BBR shows that it suppresses 3T3-L1 adipocyte differentiation, proliferation, and lipid buildup. These effects may occur due to a variety of molecular targets and intricate pathways, such as suppressing PPARγ and PPARα transactivations and inhibiting the mRNA and protein of early transcription factors C/EBPβ, PPARγ, and PPARα. (Huang et al., 2006). BBR also activates AMPK and stimulates PGC-1α to cause browning-related UCP1 expression (Zhang et al., 2014).

Moreover, Esculetin is a naturally occurring dihydroxycoumarin primarily obtained from the trunk bark and twig skin of the Chinese plant *Fraxinus rhynchophylla* Hance (Zhang et al., 2022). Esculetin (6,7-dihydroxy-2H-1-benzopyran-2-one) exerts its actions by inhibiting cell proliferation, inducing apoptosis in both preadipocytes and mature adipocytes, in addition to inhibiting adipogenesis of 3T3- L1 preadipocytes. A study found that esculetin inhibited 3T3- L1 adipocyte differentiation by suppressing the accumulation of lipids in a dose-dependent manner (Yang et al., 2006). Similarly, delphinidin, an anthocyanin present in pigmented fruits and vegetables, inhibits adipogenesis by suppressing adipogenesis and lipogenesis markers, inhibiting lipid accumulation, and increasing the expression of fatty acid metabolism genes as illustrated *in vitro* by Park M et al. (Park M. et al., 2019). Furthermore, baicalein, one of the primary flavonoids found in *Scutellaria baicalensi*s (Chinese Skullcap), has been linked to many biological activities. The capacity of baicalein to increase the expression of cyclooxygenase-2 (COX-2), which is generally downregulated during adipogenesis, may explain its anti-adipogenic activity (Cha et al., 2006).

The perennial herb *Alchemilla monticola* Opiz (ALM) has been traditionally used to treat inflammatory diseases, wounds, and burns (Tasić-Kostov et al., 2019). *Alchemilla* species are recognized chemically for their flavonoids, flavonoid glycosides, phenolic acids, and tannins (Choi et al., 2018). Remarkably, it was demonstrated that ALM extracts inhibited adipogenesis by interfering with the PI3K/AKT signaling pathway, thereby inhibiting the adipogenic markers PPARγ, C/EBPα and adiponectin (Mladenova et al., 2021). Another naturally occurring anthraquinone with a wide range of beneficial pharmacological applications is Rhein (Wu et al., 1996). It is isolated from rhizomes of medicinal plants such as Rheum undulatum, Rheum palmatum, as well as in Cassia reticulata (Huang et al., 2021). In 3T3-L1 cells, PPARγ and C/EBPα protein and mRNA levels produced by differentiation medium were found to be dramatically downregulated by rhein (Liu et al., 2011). In a recent study, rhein treatment in the mitotic clonal expansion stage has slowed down 3T3-L1 differentiation with much fewer lipid droplets accumulation and adipocyte gene expression (Huang et al., 2021). It was also found that Rhein suppresses 3T3-L1 adipocyte development in a dose- and time-dependent way.

Furthermore, turmeric as a commonly used spice in Asian cuisines (*Curcuma longa* Linn.), contains curcumin (also known as diferuloylmethane). Curcumin suppresses adipogenesis and downregulates the expression of PPARγ in 3T3-L1 adipocytes by activating AMPK (Lee et al., 2009). Curcumin decreased the activation of MAPK signalling pathways (including ERK, JNK, and p38), which prevented the differentiation of 3T3-L1 cells into adipocytes. Curcumin also restored nuclear translocation of the essential Wnt signaling component β-catenin during differentiation in a dose-dependent manner. The differentiation-stimulated production of CK1α, GSK-3β, and Axin, was inhibited by curcumin, all of which are components of the destruction complex that targets β-catenin; consequently, it blocks adipogenesis in 3T3-L1 adipocytes (Ahn et al., 2010). In another study, it was found that in inguinal WAT, curcumin increased the plasma norepinephrine levels and the expression of the β3AR gene (Wang S. et al., 2015). Both the rapid stimulation of already-existing beige adipocytes and the differentiation of beige adipocytes from their precursors depend on the release of norepinephrine at sympathetic terminals in the adrenal medulla and WAT (Bartelt and Heeren, 2014). Norepinephrine is the primary regulator of adaptive thermogenesis in brown and beige adipose tissues by upregulating PGC-1α and UCP1 expression through β-adrenergic (mainly β3) and cAMP-dependent pathways (Nedergaard and Cannon, 2014). Thus, curcumin has the potential to induce WAT browning through the norepinephrine- β3AR pathway. For a complete summary for the Plant-derived active compounds, source, and mechanism of phytochemicals responsible for obesity management, see Table 1.

**TABLE 1:** Plant-derived active compounds, source, and effects of phytochemicals responsible for obesity management.

Active compound	Source (plant species)	Effects/Mechanism of action	IC_50_ value	Dosages employed	Study type
Caffeine	*C. arabica* L. and *C. canephora* Pierre (Ferreira et al., 2019)	- Metabolic stimulant	-	100 mg/day of caffeine	Clinical trial (Dulloo et al., 1989)
		- Thermogenic agent (Diepvens et al., 2007b)			
Ephedrine	*Ephedra sinica* (Saper et al., 2004)	-Metabolic stimulant	-	1.5 mg/kg/day of ephedrine	Clinical trial (Carey et al., 2015)
		- Thermogenic agent (Stohs and Badmaev, 2016)			
Epigallocatechin gallate (EGCG)	*Camellia sinensis* L. (Thitimuta et al., 2017)	- Enhance fatty acid mobilization and oxidation (Jeukendrup and Randell, 2011)	0.45 mg/mL (Lipase Inhibition) (Jamous et al., 2018)	576 mg/day of catechins	Clinical trial (Matsuyama et al., 2008)
		- Promote browning markers (Li et al., 2021			
		- Inhibit adipogenesis (Lin et al., 2005)			
Capsaicin	*Capsicum annuum* L. (Barceloux, 2009)	-Stimulates thermogenesis Reinbach et al. (2010)	-	1 g/day of red peppers containing 1995 µg capsaicin, 247 µg nordihydrocapsaicin and 1,350 µg dihydrocapsaicin	Clinical trial (Ludy and Mattes, 2011) (Dömötör et al., 2006)
		- Enhance insulin sensitivity, and increase fat oxidation (Aryaeian et al., 2017)		- 400 µg/day of capsaicin	
		-Decreases PPARγ, C/EBPα and leptin protein expression in 3T3-L1 adipocytes (Hsu and Yen, 2007)			
Korean pine nut-free fatty acids (FFA)	*Pinus Koraiensis* (Wolff et al., 2000)	-Releases cholecystokinin (CCK), thus, enhancing satiety and reducing appetite (Pasman et al., 2008)	-	3 g of Korean pine nut FFA.	Clinical trial (Pasman et al., 2008)
Hydroxycitric acid (HCA)	*Garcinia cambogia* (S. E Ohia et al., 2002)	Enhances 5-HT release (Ohia et al., 2001)	-	2800 mg/day of HCA.	Clinical trial (Preuss et al., 2004)
Cathine (D-nor-pseudoephedrine) and cathinone (1-aminopropiophenone)	*Catha edulis* (Lemieux et al., 2015)	Increases dopamine in the brain by acting on the catecholaminergic synapses (Patel, 2000)	-	- 20 mg/kg/day of khat extract	- *In vivo* trial (Hesham et al., 2011)
				−200 and 400 g of fresh khat chewed for 4 h	- Clinical trial (Walid et al., 2006)
P57 molecule (oxypregnane steroidal glycoside)	*Hoodia gordonii* (Jain and Singh, 2013)	Increases ATP in hypothalamic neurons (MacLean and Luo, 2004)	-	100–150 mg/kg/day of *Hoodia gordonii* extract	*In vivo* trial (Jain and Singh, 2013)
Pregnane glycosides (Schneider et al., 1993)	*Caralluma adscendens* var. *Fimbriata* (Wall.) Gravely & Mayur (Dutt et al., 2012)	Downregulation of ghrelin production and neuropeptide Y (NPY) (Gardiner et al., 2005; Komarnytsky et al., 2013)	-	1 g/day of C. Fimbriata extract	Clinical trial (Rao et al., 2021b)
Forskolin	*Coleus forskohlii* Briq (Astell et al., 2013)	Increase thermogenesis and lipogenesis (Godard et al., 2005)	-	250 mg of 10% forskolin extract twice a day	Clinical trial (Godard et al., 2005)
Beta-carotenes, γ-linolenic acid, and phenols	*Stellaria medium* (Linn.) Vill. (Sharma, 2003)	Inhibits pancreatic α-amylase and lipase. (Rani et al., 2012a)	−3.71 mg/mL (Lipase inhibition)	900 mg/kg/day of *Stellaria medium* extract	*In vivo* trial (Rani et al., 2012a)
			−4.53 mg/mL (α-amylase inhibition)		
Saponins, flavonoids, and phenols	*Achyranthes aspera* (Hariharan and Rangaswami, 1970)	Inhibits lipase and α-amylase activity (Rani et al., 2012b)	−2.34 mg/mL (lipase inhibition)	900 mg/kg/day of *Achyranthes aspera* extract	*In vivo* trial (Rani et al., 2012b)
			−3.83 mg/mL (α -amylase inhibition)		
Megastigmanes and alkaloids such as *trans*-N-coumaroyltyramine, *trans*-N-feruloyltyramine, roemerine oxide, liriodenine, and annuionone D	*Nelumbo nucifera* Gaertn	-Inhibits lipase and α-amylase activity	−0.46 mg/mL (Lipase inhibition)	20 g (i.e., 10% yield) of a dark brown, *Nelumbo nucifera* leaves extract in high fat diet per day	*In vivo* and *In vitro* trial (Ono et al., 2006b)
		-Suppresses adipocyte differentiation (Ahn et al., 2013)	−0.82 mg/mL (α-amylase inhibition)		
Saponin, sapogenins and Phenanthrenes such as dioscin and diosgenin	*Dioscorea nipponica* Makino	-Suppressed blood triacylglycerol level	5–10 μg/mL (Lipase inhibition)	20–50 g/kg/day of methanol extract of Dioscorea nipponica Makino powder (DP)	*In vivo* trial (Kwon et al., 2003a)
		-Inhibits fat absorption (Kwon et al., 2003a)			
Saponins known as platycosides such as platycodin A, C, D, and deapioplatycodin D	*Platycodon grandiflorum*	-Reduces hyperlipidemia, diabetes and inflammatory diseases	-	−500 μg/mL/day of total saponin	*In vitro* trial (Xu et al., 2005)
		-Suppresses hypercholesterolemia and hyperlipidemia by inhibiting intestinal absorption of dietary fats mediated by pancreatic lipase inhibition. (Xu et al., 2005)		−500 μg/mL/day of platicodin	
Licochalcone A and Glycyrrhizin	*Glycyrrhiza uralensis*	- Inhibits pancreatic lipase activity (WonLicochalcone et al., 2007)	35 μg/mL	2, 4, and 6 mg/ml/day of licochalcone A	*In vitro* trial (WonLicochalcone et al., 2007)
		- Contributes in browning of inguinal white adipose tissue (Lee et al., 2018)			
Ursolic acid (Bacanli et al., 2019)	*Malus pumila, Ocimum basilicum, Vaccinium macrocarpon, Olea europaea, Origanum vulgare, Rosmarinus officinalis, Salvia, and Thymus* (Ikeda et al., 2008)	-Enhances insulin sensitivity and glucose intolerance (Jang et al., 2009)	-	0.5 g/kg/day of ursolic acid	*In vivo* (Jang et al., 2009)
Cinnamic acid	*Cinnamomum cassia* L.) J.Presl, *Vitis vinifera* L., *Camellia sinensis* L.) Kuntze, *Theobroma cacao* L., *Spinacia oleracea* L., *Apium graveolens* L. (Guzman, 2014)	-Improves hyperglycemia and hyperlipidemia. -Increases insulin sensitivity, and lowers blood and hepatic lipids (Kim and Choung, 2010)	-	200 mg/kg/day after of cinnamon extract	*In vivo* (Kim and Choung, 2010)
Resveratrol	*Polygonum cuspidatum*, *Veratrum grandiflorum*, *Vitis vinifera*, *Arachis hypogaea*, and *Vaccinium oxycoccos*	-Improve insulin sensitivity	-	250 mg/day of resveratrol	Clinical trial (Bhatt et al., 2012)
		-Enhance GLUT4 translocation			
		-Reduce oxidative stress			
		-Regulate carbohydrate metabolizing enzymes			
		-Activate SIRT1 and AMPK			
		-Reduce adipogenic genes. (Bagul and Banerjee, 2015)			
Procyanidins	*Prunus domestica, Prunus salicina, Malus domestica, Vitis vinifera, Prunus amygdalus,* and *Cicer arietinum*	-Enhances Insulin sensitivity	-	850 mg flavan-3-ols and 100 mg isoflavones per day	Clinical trial (Curtis et al., 2012)
		-Increases the active GLP-1 levels and controls glucose homeostasis. (González-Abuín et al., 2015)			
Mangiferin (xanthonoid)	Mango and rhizomes of *Anemarrhena asphodeloides* (Miura et al., 2001)	-Improve insulin sensitivity	-	20 mg/kg/day of mangiferin	*In vitro* (Saleh et al., 2014)
		-Altered lipid profiles			
		-Reduce the number of adipokines. (Saleh et al., 2014)			
Ginger extracts such as gingerols, shogaols, paradols, and gingerenone A	*Zingiber Officinale* (Khandouzi et al., 2015)	-Improve insulin sensitivity and glucose uptake (Sekiya et al., 2004)	-	- 30–1,000 µM/day of 6-gingerol (insulin sensitivity)	-In vitro (Sekiya et al., 2004)
		-6-gingerol decreases PPARγ, C/EBPα, and FABP4 expression and increases adiponectin expression (Cheng et al., 2022)		−25 mg/kg/day of 6-gingerol (increases adiponectic expression)	-In vivo (Cheng et al., 2022)
Saffron extracts including; carotenoids, crocin, and its aglycone crocetin, picrocrocin, and safranal	*Crocus sativus* Linn. (Kang et al., 2012)	-Insulin sensitivity enhancement	-	2.5 μg/ml/day of 47% (w/w) of saffron stigma extract	*In vitro* (Kang et al., 2012)
		-Stimulation of insulin signaling pathways			
		-Improvement of β-cell activities			
		-Promotion of glucose transporter type 4 (GLUT-4) expression			
		-Regulation of oxidative stress			
		-Repression of inflammatory pathways (Mobasseri et al., 2020b)			
Amorfrutin derivatives	Fruits of *Amorpha fruticosa*, and the edible roots of *Glycyrrhiza foetida* (Weidner et al., 2012)	-Lipid and glucose metabolism	51 nM	100 mg/kg/day of amorfrutin 1	*In vivo* (Weidner et al., 2012)
		-Improve insulin sensitivity			
		-They are potent PPARγ agonists			
		-Improve insulin sensitivity and regulate adipokines regulation. (Malapaka et al., 2012)			
Steroidal saponins called Ginsenosides	Genus *Eleutherococcus* and genus *Panax* (Vogler et al., 1999)	-Increase insulin production	-	6 g/day of Panax ginseng extract	Clinical trial (Vuksan et al., 2008)
		-Preserve pancreatic islets			
		-Increase insulin sensitivity			
		-Stimulate glucose uptake (Xie et al., 2011)			
		-Elevate the UCP1, PRDM16, and PGC-1 in 3T3-L1 cells and primary white adipocytes			
Steroidal sapogenins such as diosgenin, furostanol glycosides, alkaloids such as trigocoumarin, nicotinic acid, trimethyl coumarin, and trigonelline	*Trigonella foenum-graecum* L. (Gupta et al., 2001)	-Increase insulin release	-	50 mg/kg/day of active hypoglycemic principle isolated from water extract of seeds of Trigonella foenum graecum	*In vivo* (Puri et al., 2002)
		-Improve insulin sensitivity (Puri et al., 2002)			
		- Increase the expression of BAT signature proteins including PGC-1α, PRDM16, and UCP1 in 3T3-L1 white adipocytes (Choi et al., 2021)			
Allicin, ajoene, dithiins, allyl methyl trisulfide, diallyl sulfide, diallyl disulfide, diallyl trisulfide, and β-carolide alkaloids	*Allium Sativa*	-Increase insulin secretion and enhance insulin sensitivity (Liu et al., 2005)	-	−100 mg/kg every other day of gavage garlic oil	*In vivo* (Liu et al., 2005)
		-Increased phosphorylation on AMPK and its downstream proteins		−40 mg/kg every other day of diallyl trisulfide	
		-β-carboline alkaloid suppressed the differentiation of adipocytes			
Camphor, eugenol, cinnamaldehyde, cinnamic acid, cinnamyl alcohol, and coumarin	*Cinnamomum zeylanicum* and *Cinnamon cassia* (Hariri and Ghiasvand, 2016)	-Increase insulin sensitivity (Couturier et al., 2010)	-	−20 g/kg/day of *Cinnamon cassia* powder (Improve insulin sensitivity)	*In vivo* trial (Couturier et al., 2010)
		- Increase the expression of UCP1 and other brown adipocyte markers in 3T3-L1 adipocytes and subcutaneous adipocytes		−80 μg/ml/day of cinnamon extract (increase brown adipocyte markers	-In vivo, *ex vivo*, *in vitro* trial (Kwan et al., 2017)
		-Increase the biogenesis of mitochondrial protein (Kwan et al., 2017)			
Hypericin, hyperforin, and adhyperforin (Gray et al., 2000)	*Hypericum perforatum* L. (Tokgöz and Altan, 2020)	-Improves hyperinsulinemia, hyperglycemia, insulin tolerance, and glucose infusion rate (GIR) (Tian et al., 2015)	-	50 mg/kg/day and 200 mg/kg/day ofH. perforatum L*.* extracts	*In vitro* trial (Tian et al., 2015)
Rhein (anthraquinone)	Rheum undulatum, Rheum palmatum, and Cassia reticulata (Huang et al., 2021)	Decrease in PPARγ, C/EBPα protein and mRNA levels in 3T3-L1 cells (Liu et al., 2011)	-	2.5, 5, and 10 µM/day of rhein	*In vitro* trial (Liu et al., 2011)
Genistein	*Genista tinctoria* (Mukund et al., 2017)	Reduces PPARγ and downregulated adipogenesis (Feng et al., 2016)	-	100 µM/day of genistein	*In vitro* trial (Harmon et al., 2002)
Esculetin	*Fraxinus rhynchophylla* Hance (Zhang et al., 2022)	-Inhibits cell proliferation	-	100 and 200 µM/day of esculetin	*In vitro* trial (Yang et al., 2006)
		-Induces apoptosis in preadipocytes and mature adipocytes			
		-Inhibits adipogenesis in 3T3-L1 preadipocytes. (Yang et al., 2006)			
Berberine	*Cortidis rhizome* (Huang et al., 2006)	-Suppresses the 3T3-L1 adipocyte differentiation, proliferation, and lipid buildup (Huang et al., 2006)	-	1.25, 2.5, and 5 μM/day of berberine (Inhibit 3T3-L1 adipocyte differenciation)	*In vitro* trial (Huang et al., 2006)
		- Increases the expression of browning-related UCP1 (Zhang et al., 2014)			
Baicalein	*Scutellaria baicalensi*s (Cha et al., 2006)	Increase the expression of COX-2 (Cha et al., 2006)	-	100, 150 and 200 μM/day of baicalein	*In vitro* trial (Cha et al., 2006)
Flavonoids, glycosides, phenolic acids, and tannins (Choi et al., 2018)	*Alchemilla monticola* Opiz. (ALM) (Tasić-Kostov et al., 2019)	Inhibits the adipogenic markers PPARγ, C/EBPα, and adiponectin (Mladenova et al., 2021)	-	5, 10 and 25 μg/ml/day of A. monticola extract	*In vitro* trial (Mladenova et al., 2021)
Delphinidin	Pigmented fruits and vegetables	-Inhibit the production of adipogenesis and lipogenesis markers	-	25, 50 and 100 µM of Delphinidin-3-O-β-Glucoside	*In vitro* trial (Park et al., 2019b)
		- Increase the expression of fatty acid metabolism genes in 3T3-L1 adipocytes. (Park et al., 2019b)			
Curcumin	Turmeric (*Curcuma longa* Linn) (Lee et al., 2009)	-Activate the AMP-activated protein kinase (AMPK) in 3T3-L1 cells (Lee et al., 2009)	-	−10–50 µM/day of curcumin (Activate AMPK in 3T3-L1 cells)	- *In vitro* trial (Lee et al., 2009)
		-Increases the plasma norepinephrine levels and the expression of the β3AR gene (Wang et al., 2015b)		- 50 or 100 mg/kg/day of curcumin (Increase plasma NE levels)	-In vivo trial (Wang et al., 2015b)

## 4 Safety considerations and limitations of natural products for weight management

Despite the previously demonstrated benefits of plant-derived natural products in weight management with minimal side effects, it is important to acknowledge that isolated phytochemicals and plant extracts can occasionally impose a potential risk on human health. While these substances hold promise as therapeutic agents, their inherent complexity and diverse chemical composition may give rise to unintended consequences when administered in concentrated forms. For instance, despite the short-term increase in thermogenesis associated with caffeine consumption, long-term studies involving obese subjects have shown that it does not lead to greater weight loss compared to a placebo. Interestingly, when a group with habitual high caffeine intake (approximately 300 mg/day) received a combination of green tea and caffeine, there was no significant improvement in body weight maintenance after weight loss, suggesting a potential loss of sensitivity to caffeine over time (Westerterp-Plantenga, 2010b). While caffeine has been extensively studied, there is limited research focusing specifically on its long-term effects as an anti-obesity treatment. Most studies have primarily explored short-term effects or specific metabolic changes. Therefore, the efficacy and safety of using caffeine as a long-term anti-obesity strategy remain uncertain. Ephedrine, is another stimulant utilized as an anti-obesity agent for its metabolism-enhancing properties, is accompanied by various limitations and concerns. Its administration can lead to noteworthy cardiovascular side effects, such as elevated heart rate and blood pressure, and has been linked to an augmented risk of heart attack, stroke, and withdrawal symptoms upon abrupt cessation. Consequently, due to these safety concerns, the utilization of ephedrine as a weight loss aid is not recommended, resulting in its prohibition or restriction in numerous countries (Gadde and Atkins, 2020). Additionally, a notable limitation linked to capsaicin is the difficulty of achieving a significant weight loss effect solely through dietary intake, due to the challenge of attaining an adequate dosage. Furthermore, at anti-obesity doses, capsaicin possesses the potential to cause gastrointestinal discomfort in specific individuals, thereby limiting its practical utilization (Li et al., 2020).

Differently, appetite suppressants also show limitations on the long-run use. Although khat usage is prevalent, psychosis cases were reported. However, it has been revealed about some cases of psychotic reactions in Somalian males, emphasizing the significance of recognizing the medical and psychiatric complications of khat abuse. Notably, the side effects of khat, such as gastrointestinal discomfort, and its potential involvement in forensic cases of homicide and combined homicide-suicide, warrant concern. Cultural dislocation may further amplify the adverse effects of khat consumption. Similar to other well-known amphetamine-type stimulants, chronic use of synthetic cathinone compounds can significantly impact the central nervous system, leading to acute psychosis, hypomania, paranoid ideation, and delusions (Pantelis et al., 1989; Weinstein et al., 2017). Despite its limited availability and high public demand, *Hoodia gordonii* has been associated with potential side effects including skin reactions, elevated heart rate, and high blood pressure. However, the main focus of scientific studies has been on quality control due to the scarcity of *H. gordonii* plant material resulting from its sparse geographical distribution, slow maturation rate, and the requirement of permits for cultivation and export. The high demand has led to a significant risk of product adulteration (Vermaak et al., 2011).

Both garlic and fenugreek have a history of safe medicinal use for most individuals. Potential side effects of garlic include gastrointestinal discomfort, sweating, dizziness, allergic reactions such as (allergic contact dermatitis, generalized urticaria, angiedema, pemphigus, anaphylaxis and photoallergy), and bleeding (Borrelli et al., 2007), whereas anecdotal reports suggest less serious side effects such as diarrhea and indigestion for fenugreek. Additionally, the aqueous extract of fenugreek has been shown to inhibit the coagulation process *in vitro*, resulting in a significant prolongation of prothrombin time and inhibition of clot formation (Taj Eldin et al., 2013). Thus, combining both plants and/or extract for the purpose of reducing overweight could pose a bleeding adverse reaction. On the same context, clinical and experimental studies provide evidence supporting the antidepressant-like effects of St. John’s Wort due to a non-selective blockade of the reuptake of serotonin, noradrenaline and dopamine. However, it is important to acknowledge the side effects associated with its use, including nausea, rash, fatigue, restlessness, photosensitivity, acute neuropathy, and even episodes of mania and serotonergic syndrome when used concurrently with other antidepressant drugs. These side effects indicate that *H. perforatum* extracts may possess significant pharmacological activity by targeting various neurotransmission systems involved in depression. Nevertheless, there is limited information available regarding the safety of *H. perforatum*, including potential interactions with other medications (Rodríguez-Landa and Contreras, 2003).

Cumulative evidence suggests that under specific conditions, ursolic acid may exhibit adverse reactions, including cytotoxicity. Furthermore, the therapeutic application of ursolic acid is significantly hindered by its poor solubility in aqueous medium and limited bioavailability *in vivo*. Notably, ursolic acid has been observed to induce inflammation by enhancing the production of nitric oxide and TNF-α in resting macrophages through NF-κB transactivation, and upregulates IL-1β, an important pro-inflammatory mediator. In the context of male rat spermatogenesis, the extract of ursolic acid was found to impede the physiological maturation of sperm, evident by decreased sperm count and motility, potentially attributed to the depletion of testosterone at the target level (Sun et al., 2020). Finally, obesity is a multifactorial condition that requires a comprehensive approach for effective management. Relying solely on single plant extract or one phytochemical component without addressing multi-approaches, such as appetite suppression, lipases inhibition, insulin sensitivity enhancement, in addition to other mechanisms, is unlikely to lead to sustainable weight loss. For instance, the weight-loss supplement Buginawa (Bugi) contains twelve different medicinal herbs in a novel water extract combination that improves insulin sensitivity, decreases PPARγ, and C/EBPα, and inhibits adipogenesis (Park Y.-J. et al., 2019).

## 5 Conclusion

Recently, obesity affects much more than just physical appearance and is now recognized as a medical condition that should be treated to minimize the risk of developing other metabolic diseases like diabetes. The mechanisms exploited by marketed anti-obesity medicines are appetite suppression by increasing norepinephrine, dopamine, and serotonin in the synaptic clefts, pancreatic lipase, and amylase inhibition, and gastric emptying slowing. However, the occurrence of unwanted, adverse effects affecting cardiovascular, psychological, and gastrointestinal systems urge for more effective treatment plans with minimal risk/complications. Phytochemicals derived from plants seems as a promising source of anti-obesity drugs as majority of them confer benefits via the same mechanisms with fewer adverse events. Exceptionally, some natural products also display other important features that could be essential as anti-obesity drugs, especially WAT browning, thermogenesis and adipocyte apoptosis induction, and adipogenesis inhibition. At least so far, those mechanisms have not been shown by any synthetic anti-obesity agents. Mechanisms explored in natural products are; metabolic and thermogenic stimulation, appetite regulation, pancreatic lipase and amylase inhibition, insulin sensitivity enhancement, and adipogenesis inhibition and adipocyte apoptosis induction. The phytochemicals involved in fighting obesity usually affect more than one specific mechanism since the fat storage and energy expenditure processes are complicated and intricate. Uncoupling protein UCP-1, PR domain containing 16 (PRDM16), and peroxisome proliferator-activated receptor (PPAR)-γ have a role in the browning of WAT and consequently reducing lipid accumulation. Most plant extracts/isolated compounds have been found to affect more than one of these thermogenic transcriptional factors. Moreover, SIRT1 and AMPK are controlled by phytochemicals and exert their actions on the above-mentioned transcriptional factors through the induction of the β_3_ adrenergic receptor that finally induces mitochondrial biogenesis. The strategy that can effectively reduce obesity is to inhibit/activate more than one mechanism with different pathways, can be more effective in addressing obesity. Furthermore, a holistic approach that combines a balanced diet, regular exercise, behavior modifications, and medical guidance, if necessary, is essential for long-term success in weight management.
